# Age- and height-dependent bias of underweight and overweight assessment standards for children and adolescents

**DOI:** 10.3389/fpubh.2024.1379897

**Published:** 2024-04-24

**Authors:** Yosuke Isoyama, Sayaka Nose-Ogura, Mariko Jana Ijitsu, João Gabriel Segato Kruse, Narumi Nagai, Momoko Kayaba, Hitomi Ogata, Madhur Mangalam, Ken Kiyono

**Affiliations:** ^1^Graduate School of Engineering Science, Osaka University, Osaka, Japan; ^2^Japan High-Performance Sport Center, Department of Sports Medicine and Research, Japan Institute Sports Sciences, Tokyo, Japan; ^3^Department of Obstetrics and Gynecology, University of Tokyo Hospital, Tokyo, Japan; ^4^School of Human Science and Environment, University of Hyogo, Himeji, Japan; ^5^Faculty of Medicine, University of Tsukuba, Tsukuba, Japan; ^6^Graduate School of Humanities and Social Sciences, Hiroshima University, Hiroshima, Japan; ^7^Division of Biomechanics and Research Development, Department of Biomechanics, and Center for Research in Human Movement Variability, University of Nebraska at Omaha, Omaha, NE, United States

**Keywords:** body mass index (BMI), height dependence, international obesity task force (IOFT), Japanese standards, overweight, underweight

## Abstract

**Background:**

Precision in evaluating underweight and overweight status among children and adolescents is paramount for averting health and developmental issues. Existing standards for these assessments have faced scrutiny regarding their validity. This study investigates the age and height dependencies within the international standards set by the International Obesity Task Force (IOTF), relying on body mass index (BMI), and contrasts them with Japanese standards utilizing the percentage of overweight (POW).

**Method:**

We scrutinized a comprehensive database comprising 7,863,520 children aged 5–17 years, sourced from the School Health Statistics Research initiative conducted by Japan's Ministry of Education, Culture, Sports, Science, and Technology. Employing the quantile regression method, we dissected the structure of weight-for-height distributions across different ages and sexes, quantifying the potentially biased assessments of underweight and overweight status by conventional criteria.

**Results:**

Applying IOFT criteria for underweight assessment revealed pronounced height dependence in males aged 11–13 and females aged 10–11. Notably, a discernible bias emerged, wherein children in the lower 25th percentile were classified as underweight five times more frequently than those in the upper 25th percentile. Similarly, the overweight assessment displayed robust height dependence in males aged 8–11 and females aged 7–10, with children in the lower 25th percentile for height deemed obese four or five times more frequently than their counterparts in the upper 25th percentile. Furthermore, using the Japanese POW criteria for assessment revealed significant age dependence in addition to considerably underestimating the percentage of underweight and overweight cases under the age of seven. However, the height dependence for the POW criterion was smaller than the BMI criterion, and the difference between height classes was less than 3-fold.

**Conclusion:**

Our findings underscore the intricacies of age-dependent changes in body composition during the growth process in children, emphasizing the absence of gold standards for assessing underweight and overweight. Careful judgment is crucial in cases of short or tall stature at the same age, surpassing sole reliance on conventional criteria results.

## 1 Introduction

Child and adolescent thinness correlates with a range of adverse outcomes, including stunted growth (1–3), delayed maturation (1, 4), nutritional deficiencies (5, 6), and diminished cognitive capability (7, 8). This condition also heightens the risk of osteoporosis (9, 10), weakens the immune system (11, 12), leads to anemia (13, 14), respiratory failure (15, 16), and gives rise to complications in wounds (17, 18). In women, it contributes to infertility (19, 20), preterm birth (21–24), and an elevated risk of all-cause mortality (25–27). Conversely, obesity is linked to high blood pressure (28–30), elevated cholesterol levels (31, 32), type 2 diabetes (33–35), respiratory issues like asthma and sleep apnea (36–38), and the early onset of joint problems (39–41). Since childhood and adolescent thinness and obesity amplify morbidity and mortality risks later in life, precise assessment of these conditions is paramount for effective prevention and management. It is no surprise that evaluating thinness, underweight, overweight, and obesity status has become a cornerstone in numerous school health and medical care institutions. However, a common practice among practitioners in these fields is the routine application of traditional criteria such as the body mass index (BMI) to assess the growth and nutritional status of children and adolescents (42–48).

BMI, calculated as weight in kilograms divided by height in meters squared, has long served as a widely used metric for assessing body thinness and fatness across all age groups, including children and adolescents. Over the past five decades, entities such as the World Health Organization (WHO), insurance companies, medical professionals, nutritionists, and elementary schools have employed some BMI-based criteria to assess thinness, underweight, overweight, and obesity status (42, 49–52). BMI has been established as a body mass assessment measure with almost no height dependence (53). Notably, it has been confirmed that the correlation between BMI and height is almost zero in adults (54).

However, persistent objections have been raised regarding the suitability of BMI for pediatric populations. Concerns include its failure to account for variations across race/ethnic groups (55–58), sex (57, 59–61), and age spans (57, 62, 63). Furthermore, critics argue that BMI is an imperfect measure of body fat content, as it does not consider factors like muscle mass, bone density, and overall body composition (64–66). Moreover, there is skepticism about the direct association between increased BMI and heightened cardiovascular and mortality risks (67). In this context, we have recently posed a fundamental question about the allometric uni-scaling relationship that typically links weight and height in children and adolescents (68). Specifically, we analyzed a large-scale Japanese database encompassing 7,863,520 children aged 5–17 using a newly introduced method to test scaling properties through quantile regression (68). Our investigation revealed remarkable multi-scaling properties in males aged 5–13 and females aged 5–11, converging to uni-scaling as they approached 17 years for both sexes, exhibiting a scaling exponent close to 2. While we affirmed the suitability of conventional BMI as an objective height-adjusted mass measure around 17 years, nearing adulthood, for both males and females, its validity was not confirmed in younger age groups. Our findings shed light on the complexity of children's weight-for-height relation, challenging the assumption of a simplistic growth pattern. Consequently, BMI-based cutoffs prove inadequate for assessing thinness and obesity in children and adolescents, emphasizing the need for more nuanced and age-specific approaches in evaluating weight status. Not surprisingly, assessing children's body structure is complex, and no gold standard method exists; improvements to evaluation methods continue to be proposed (69).

In Japan, to eliminate the height dependence of underweight and overweight status, cutoffs for obesity in schoolchildren have deviated from BMI-based criteria and instead rely on the percentage of overweight (POW) (70–73). The POW is calculated using the formula:


(1) $$\text{POW}=\left(\frac{\text{body weight}-\text{standard body weight}}{\text{standard body weight}}\right)\times 100,$$

wherein the standard body weight is calculated using a linear equation *aH*−*b* with the coefficients *a* and *b* determined by sex and age (74). POW ≤ −20% is categorized as underweight, and POW ≥20% is categorized as indicative of obesity. A recurring observation raises concerns–children classified as obese according to their POW measurement may not align with a clinical appearance of obesity. This discrepancy is particularly pronounced in the case of children with shorter stature, making POW overestimate obesity in children with diminished height (75). Also, Japanese standards utilizing the POW have demonstrated a poor correlation with adiposity (75, 76).

Recognizing the potential limitations of BMI and POW across different contexts, it becomes imperative to establish robust scientific methodologies for scrutinizing their foundations and validity. The challenges surrounding these metrics can be systematically categorized into three key domains: (i) The underlying principles of their respective formulae, (ii) The effectiveness of these metrics as precise measures of thinness and fatness across populations with varying heights, and (iii) The validity of cutoff values for nutritional status screening. Addressing these challenges requires the application of diverse scientific approaches, including a physiological methodology relying on body composition measurements, an epidemiological approach grounded in disease prevalence, and a statistical approach based on the analysis of extensive height and weight datasets. This study predominantly adopted a novel statistical approach to address these intricate issues.

The study outline unfolds as follows: we analyzed a vast database comprising 7,863,520 children aged 5–17 years, collated from the School Health Statistics Research initiative by Japan's Ministry of Education, Culture, Sports, Science, and Technology. Employing the quantile regression method, we dissected the weight-for-height distributions across various ages and sexes, quantifying the potentially skewed underweight and overweight assessments by conventional criteria. Our analysis revealed a significant height dependency in underweight and overweight assessments across different ages and genders, underscoring the nuanced age-related changes in body composition during childhood growth and highlighting the absence of universally accepted standards for underweight and overweight assessment. Thus, we advocate against adopting a one-size-fits-all metric for all ages and heights within this population. To facilitate the adoption of our methodology by clinicians and public health professionals, we provide sex- and age-specific plots (Supplementary Figures S1–S26). These plots aid in estimating a child's percentile position by mapping the measured weight and height onto the centile curve space, offering a user-friendly visual tool for interpretation and application in diverse healthcare settings. A more precise evaluation of growth and nutritional status during childhood and adolescence is anticipated to enhance public health initiatives and policies, particularly in regions where malnutrition or childhood and adolescent obesity are prevalent.

## 2 Methods

In the realm of statistical analysis, conventional methods for determining underweight and overweight status in children and adolescents typically establish assessment criteria by considering the rare occurrence of specific combinations of sex, age, weight, and height—a methodology reminiscent of the reference interval used in clinical examinations, such as the 95% central range between the 2.5th and 97.5th percentiles of a test indicator. In assessing underweight and overweight, these conventional criteria strive for a height-independent evaluation method tailored to each age and sex. Using a comprehensive demographic analysis of school-age children from Japan, the present study questions the characteristics and validity of these traditional criteria for determining underweight and overweight status in children and adolescents. Our investigation highlights a notable height dependency observed in BMI and IOTF cutoffs. We illustrate that Japan's POW (proportion of overweight) criterion exhibits a significant dependence on height and age, lacking adjustment for the probability of extreme weight occurrences. While BMI cutoffs may seem reasonable upon initial scrutiny of BMI distributions, they fail to adequately account for the age-specific height dependence of weight. Our analysis strongly underscores that the POW criterion cutoffs in Japan are markedly influenced by age, overlooking the age-related variations within the weight-height distribution and the inherent asymmetry in weight distribution.

### 2.1 Dataset: comprehensive demographic analysis of school-age children

We drew upon data from the School Health Statistics Research initiative by Japan's Ministry of Education, Culture, Sports, Science, and Technology. This nationwide survey employs a robust stratified random sampling approach, selecting schools within each prefecture based on student enrollment numbers. This ensures a representative mix of small and large institutions. Within these size strata, schools are chosen randomly for the survey, and the children included undergo a systematic sampling method, accounting for sex and age. The survey, conducted annually from April to June, traditionally captures a comprehensive snapshot of the physical development landscape. However, due to the unprecedented challenges posed by the COVID-19 pandemic 2020, the survey schedule was adapted for year-round implementation. Consequently, data collected post-2020 was not integrated into our analysis. The dataset consisted of crucial demographic factors such as sex, age, height, and weight of a staggering 7,863,520 children aged between 5–17 collected from 2008–2019. We excluded the data for 2.72% weight-for-height samples, accounting for missing values, resulting in 8,083,466 height-for-weight samples. The number of samples for each sex and age is summarized in Table 1. The utilization and analysis of the dataset were carried out with permission from the Japanese Ministry of Education, Culture, Sports, Science, and Technology. No ethical issues are associated with the use of this dataset.

**Table 1:** The number of weight-for-height samples for each age and sex after removing samples with missing values.

**Age**	**Number of samples**	
**[years]**	**Males**	**Females**
5	358,375	355,134
6	260,536	260,090
7	261,178	260,666
8	261,374	260,903
9	261,852	261,426
10	261,898	261,643
11	262,405	261,994
12	427,950	428,705
13	428,295	429,324
14	429,119	429,896
15	239,003	241,696
16	238,935	241,314
17	238,851	240,958
Subtotal	3,929,771	3,933,749
Total	7,863,520	

### 2.2 Smoothed bootstrap

We analyzed height and weight data recorded in integer values, measured in centimeters (cm) and kilograms (kg). Recognizing the limitations of discrete distributions inherent in integer-valued data derived from real-valued measurements, we applied the smoothed bootstrap technique to bolster statistical confidence (77). In this approach, rows from the dataset are randomly sampled with replacement, creating samples of size *N* from the initial dataset of size *N*. Subsequently, random noise, generated from a bivariate Gaussian kernel density, is introduced to each pair of heights and weights in the sampled row. The smoothing bandwidth is determined using a rule-of-thumb bandwidth selector for bivariate Gaussian kernels (78). This study conducted the bootstrap technique 200 times, calculating a statistical estimate for each smoothed bootstrap replication.

### 2.3 Criteria for assessing underweight and overweight status

We used BMI-based criteria established by IOFT and POW-based criteria that are widely used in Japan. The BMI cut-off points established by the IOTF were obtained from data (97,876 males and 94,851 females from birth to 25 years of age) from six countries (Brazil, Great Britain, Hong Kong, The Netherlands, Singapore, and the United States) collected between 1963 and 1993. The BMI cutoffs for adults were extended to those for younger ages so that the positions of the centiles corresponding to the 18-year-old cutoff centiles (overweight (25 kg/m^2^), obese (30 kg/m^2^), and underweight (level 1:18.5 kg/m^2^; level 2:17.0 kg/m^2^, and level 3:16.0 kg/m^2^) is maintained.

In Japan's School Health Statistics Survey, the weight-for-height distribution by sex and age is assumed to be approximated by a bivariate normal distribution. The standard weight-for-height relation is then modeled by a regression line fitted to data in its 95% probability elliptic region. This linear model calculates the standard weight for a given child's weight. Children with a Percent of Overweight (POW), defined as the percent deviation from the standard weight (as per Equation 1), of 20% or more are classified as obese, while those with a POW of –20% or less are considered underweight. The parameters of the linear model are derived from the Health Statistics Survey.

### 2.4 Quantile regression

**Figure 1:**
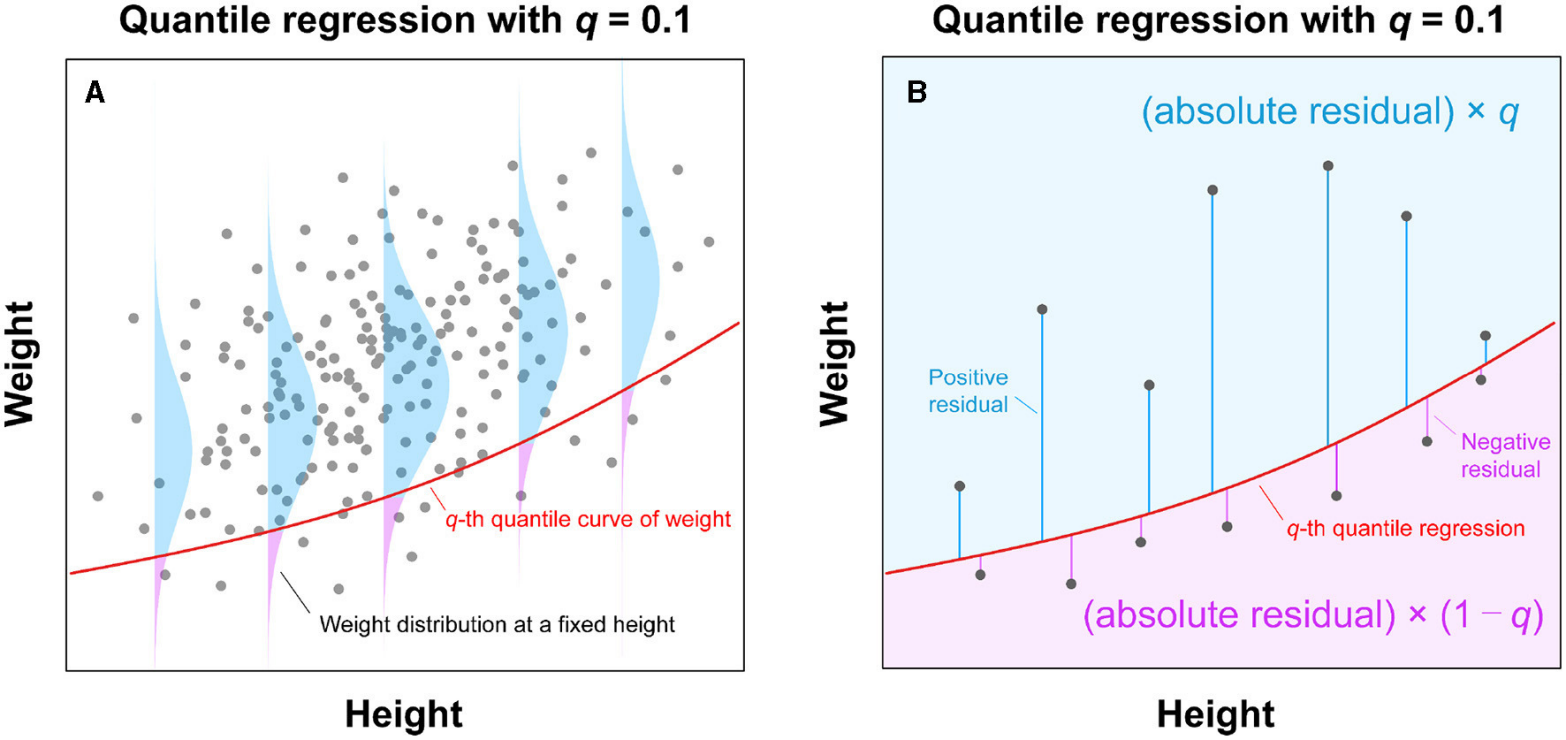
Illustration of quantile regression analysis for a weight-for-height distribution. **(A)** Using observed weight and height data, this analysis estimates a curve (*solid red line*) through the *q*th quantile point of the weight distribution for each height. **(B)** The *q*th quantile curve (*solid red line*) can be estimated by applying the asymmetric weights to the residuals from a fitted curve and minimizing the sum of weighted absolute residuals. In the case of *q* = 0.1, the negative residuals result in a nine times larger weight (i.e., 1−*q* = 0.9) than the positive ones (i.e., *q* = 0.1).

Quantile regression, a method introduced by Koenker in 1978, focuses on estimating conditional quantiles (centiles) of a response variable, thereby liberating the analysis from the assumption that variables behave uniformly across the tails of the distribution compared to the mean (79). For example, in the context of a quantile regression utilizing a cubic polynomial, the objective is to minimize the sum of weighted absolute residuals:


(2) $$\begin{array}{l} \;\sum_{i:w_{i}\geq f(h_{i};{\left\{a_{j}\right\}}_{j=0}^{3})}\;\tau |w_{i}-f(h_{i};\hat{C},\hat{\alpha })|\\ +\sum_{i:y_{i}<f(x_{i};\hat{C},\hat{\alpha })}\left(1-\tau )|w_{i}-f(h_{i};{\left\{a_{j}\right\}}_{j=0}^{3})\right|, \end{array}$$

with weight *w*_*i*_ as the response variable, *i* as the index of an individual, $f\left(h_{i};{\left\{a_{j}\right\}}_{j=0}^{3}\right)=a_{0}+a_{1}h_{i}+a_{2}h_{i}^{2}+a_{3}h_{i}^{3}$ as the cubic polynomial (Equation 2). We determined the model parameters as ${\left\{{â}_{j}\right\}}_{j=0}^{3}$. To align our analysis with the targeted quantile (*q*), we set τ equal to *q*/100 while applying the *q*-th quantile regression. A simple search algorithm (80) was employed for parameter identification (see Ogata et al. (68) for details). See Figure 1 for an illustration of quantile regression used to analyze weight-for-height distribution.

**Figure 2:**
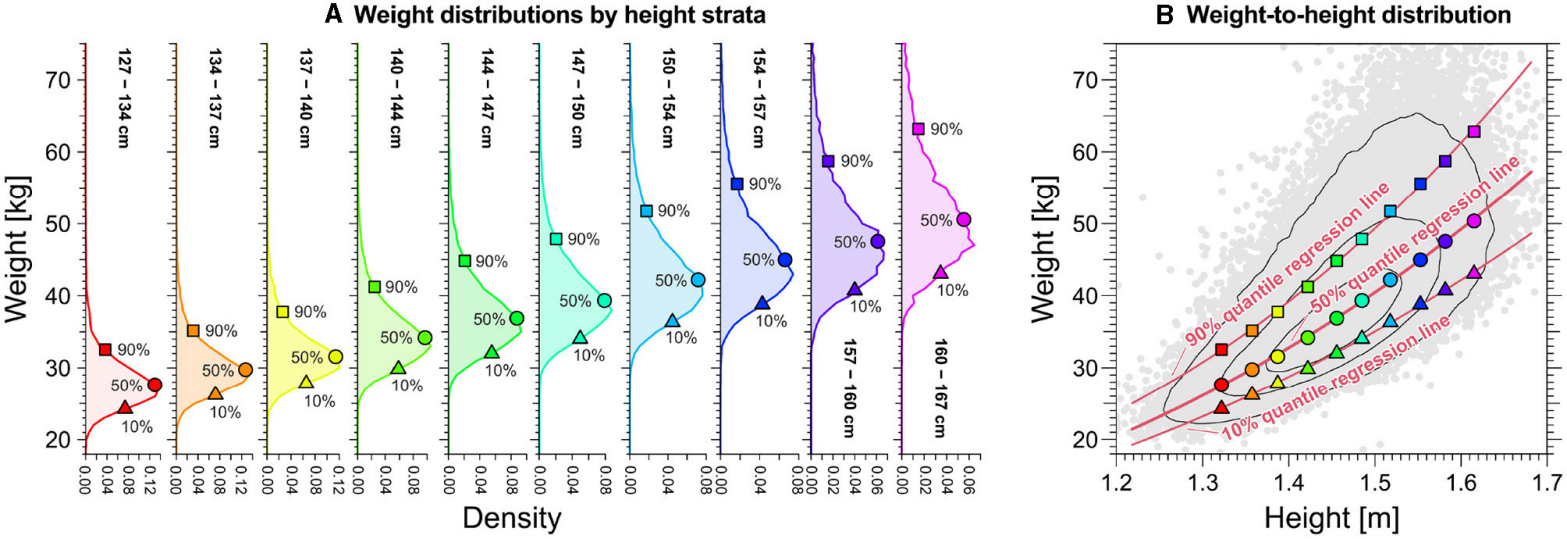
Relation between centiles of weight distributions by height strata and quantile regression line of weight-for-height distributions for the specific case of 11-year-old females. **(A)** Weight distributions by height strata. *Triangles, circles*, and *squares* denote each distribution's 10th, 50th, and 90th percentiles, respectively, for each height strata. **(B)** Quantile regression curves (*solid red lines*) using the cubic function for weight-for-height distributions. *Triangles, circles*, and *squares* denote each distribution's 10th, 50th, and 90th percentiles by height strata, respectively, shown in **(A)**.

Quantile regression yields credible estimates of centile (percentile) curves within the weight-for-height distributions (*solid lines* in Figure 2B). An alternative method for determining percentile points, involving the division of height into strata, is illustrated in Figure 2A; however, this approach fails to provide continuous weight percentile curves. In contrast, quantile regression, assuming a cubic function (*solid lines* in Figure 2B), aligns well with the height strata-based method (Figure 2A), establishing itself as a reasonable approach to estimating continuous weight percentile curves.

## 3 Results

### 3.1 Height and age dependence of underweight and overweight assessment

**Figure 3:**
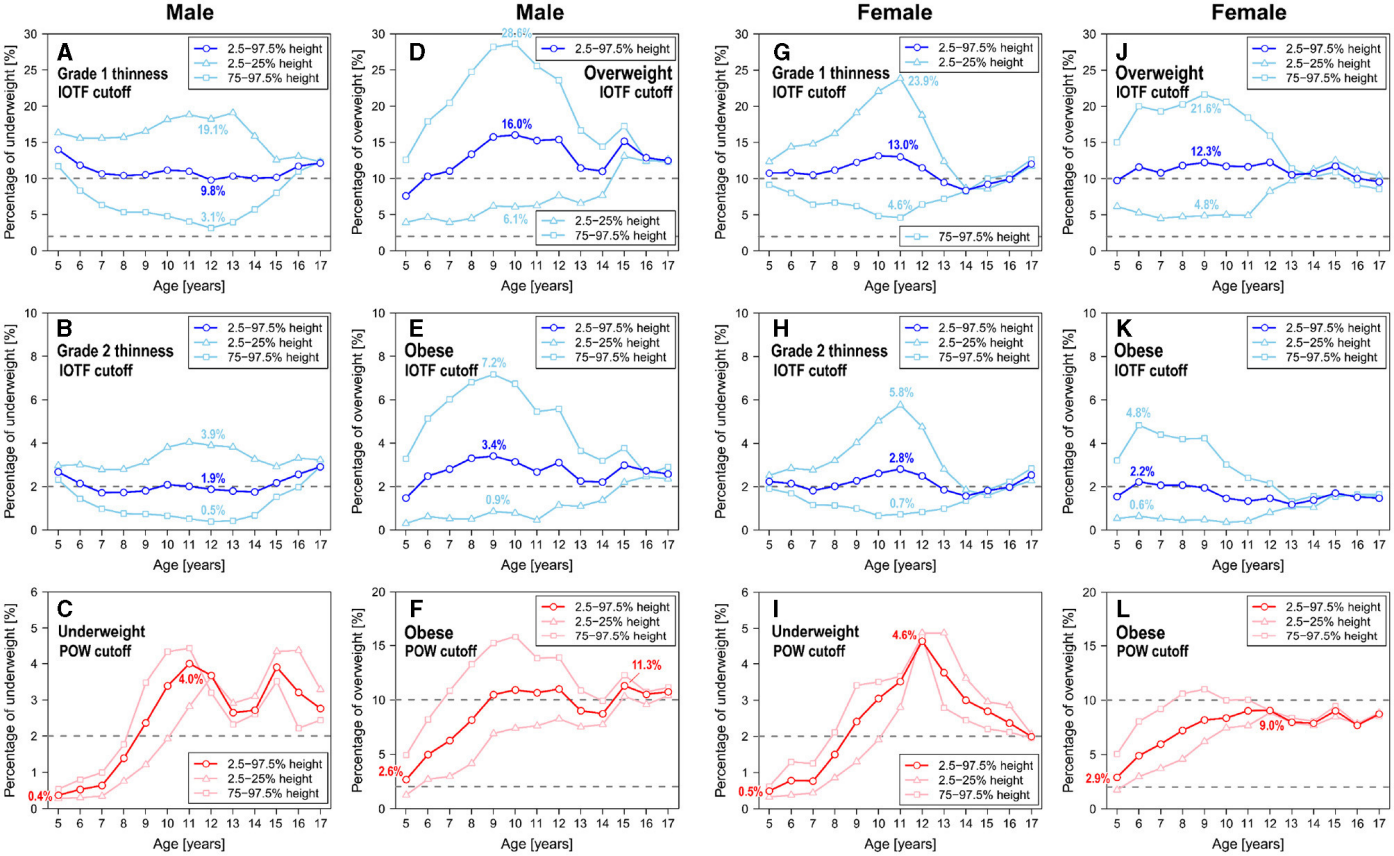
Age and height dependence of the percentages of underweight and overweight children and adolescents, based on anthropomorphic data spanning 2018. **(A)** IOFT cutoffs for grade I thinness in males. **(B)** IOFT cutoffs for grade II thinness in males. **(C)** POW cutoffs for underweight in males. **(D)** IOFT cutoffs for overweight in males. **(E)** IOFT cutoffs for obesity in males. **(F)** POW cutoffs for obesity in males. **(G)** IOFT cutoffs for grade I thinness in females. **(H)** IOFT cutoffs for grade II thinness in females. **(I)** POW cutoffs for underweight in females. **(J)** IOFT cutoffs for overweight in females. **(K)** IOFT cutoffs for obesity in females. **(L)** POW cutoffs for obesity in females.

Figure 3 presents the estimated results for the proportion of underweight and overweight individuals across sexes for specific age subgroups, ranging from the bottom 2.5–25% of height and the top 75–97.5% of height. The expectation is that, in the absence of height dependence, the three curves in each panel in Figure 3 should coincide. Notably, for males aged 16 years and older and females aged 14 years and older, the percentage of underweight and overweight cases, as per BMI standards, must remain consistent regardless of height differences, suggesting that BMI mitigates height dependence post-maturation. However, during the rapid growth phases of children and adolescents, height and age dependence persist in underweight and overweight assessments.

As indicated in Figure 3, it is noteworthy that the BMI-based IOFT criteria exhibited age ranges characterized by pronounced height dependence. Specifically, underweight assessment using the IOFT criteria exhibited a marked height dependence in males aged 11–13 (Figures 3A, B) and females aged 9–11 (Figures 3G, H), with children in the lower 25th percentile for height being designated underweight five times more often than those in the upper 25th percentile. In 12-year-old males (Figure 3A) and 11-year-old females (Figure 3H), the short subgroup exhibited an eightfold percentage of grade 2 thinness than the tall subgroup. Similarly, overweight assessment using the IOFT criteria displayed a substantial height dependence in males aged 8–11 (Figures 3D, E) and females aged 7–10 (Figures 3J, K). In this context, children in the lower 25th percentile for height were identified as obese four or five times more frequently than their counterparts in the upper 25th percentile. In 9-year-old males (Figure 3E) and 6-year-old females (Figure 3K), the tall subgroup exhibited an eightfold percentage of grade 2 thinness than the short subgroup. Comparable distinctions were noted among males and females across various age brackets, albeit with reduced magnitude. The height dependence illustrated in Figure 3 remained consistent throughout the years spanning 2008–2019.

On the contrary, POW-based assessment exhibited significant age dependence. However, variation in the percentage of underweight and obese cases across height subgroups was limited to less than threefold. The outcomes of the POW-based assessment revealed remarkably low percentages of thin and obese individuals at age 5 (Figures 3C, F, I, L), which contradicts the findings from the evaluation of the IOFT criteria. Regarding underweight assessment, the percentage for 11-year-old males was ten times higher than that for 5-year-old males (Figure 3C), with comparable disparities observed between 12-year-old females and 5-year-old females (Figure 3I). Consistent age dependence was observed throughout the period 2008–2019. Thus, this age-related pattern may indicate cutoff positions rather than accurately representing the percentage of underweight individuals.

**Figure 4:**
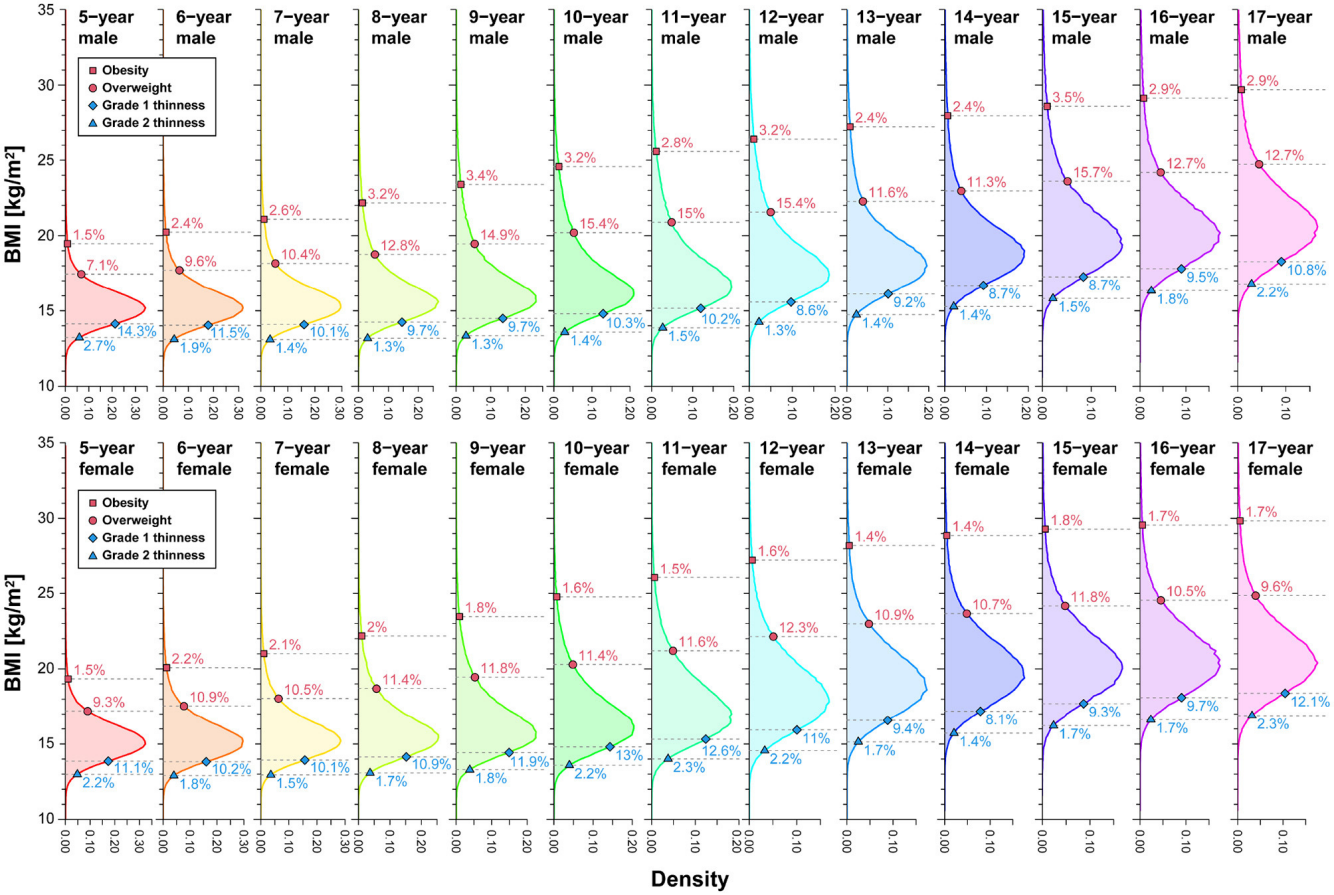
Upper- and lower-tail probabilities (percent) corresponding to IOTF cutoffs by sex and age. The values (%) in each panel denote the percentiles of each BMI distribution corresponding to the cutoffs. We analyzed data spanning 2008–2019.

Establishing BMI-based criteria for thinness and fatness in children and adolescents involves considering the tail probability of BMI within specific sex and age cohorts (42, 81). This enables the identification of potential anomalies in weight and height combinations with low observation probability. To validate this approach, we computed the BMI distribution by sex and age, delineating cutoffs for grade 1 and grade 2 thinness, overweight, and obese, as illustrated in Figure 4. The corresponding tail probabilities (percentages) for these cutoffs were also determined. In Figure 4, the upper-tail probability for obese cutoffs ranged from 1.5–3.5% for males and 1.4–2.2% for females, while the overweight cutoffs varied from 7.1–15.7% for males and 9.3–12.3% for females. Both sexes exhibited a consistent upper-tail probability of about 2% for obese cutoffs and around 10% for overweight cutoffs. The thinness assessment showed a lower-tail probability of about 10% and 2% for grade 1 thinness and grade 2 thinness cutoffs, respectively, in both males and females.

**Figure 5:**
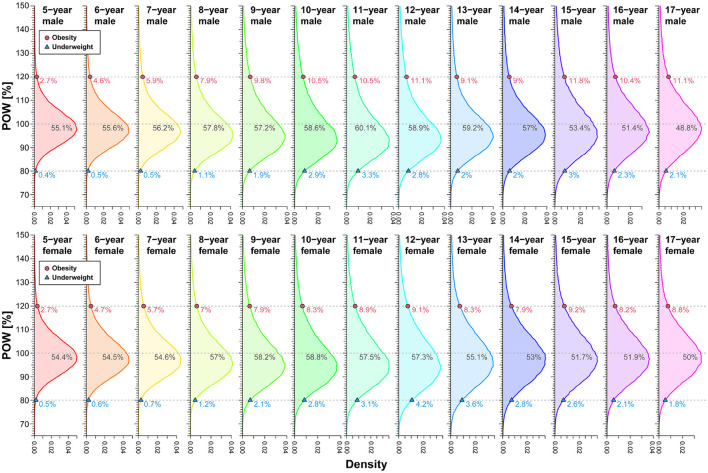
Upper- and lower-tail probabilities (percent) corresponding to Japan's POW cutoffs by sex and age. The values (%) in each panel denote the percentiles of each POW distribution corresponding to the cutoffs. We analyzed data spanning 2008–2019.

We also examined the POW distribution categorized by sex and age, outlining the cutoffs for underweight and obesity, as illustrated in Figure 5. While the age dependence of the POW distribution may seem inconspicuous initially, a closer inspection reveals significant variations in tail probabilities across age groups. We observed that, for males, the upper-tail probability of obesity ranged from 2.7–11.8%, and thinness exhibited a lower-tail probability spanning 0.4–3.3%. In females, the upper-tail probability of obesity varied from 2.7–9.2%, while thinness demonstrated a lower-tail probability ranging from 0.5–4.2%. Notably, the assessment based on the constant cutoff of POW revealed a strong age dependence in tail probability, unveiling a pronounced asymmetry in the assessment of underweight and obese status.

We scrutinized the weight-for-height distribution across sexes and age brackets to unravel the intricate relationship between age and height within traditional standards. As illustrated in Figure 6 (see also Supplementary Figures S1–S26), we employed quantile regression to derive weight percentile curves, comparing with conventional cutoffs for thinness and fatness assessment. In Figure 6B, the gray shaded areas delineate the height distribution's 50%, 90%, and 99% central regions for 17-year-old males. The proximity and parallelism between centile and cutoff curves signify minimal height dependence in the assessment process. This examination sheds light on the nuanced interplay of height and age within conventional standards, adding depth to our understanding of weight-for-height dynamics.

**Figure 6:**
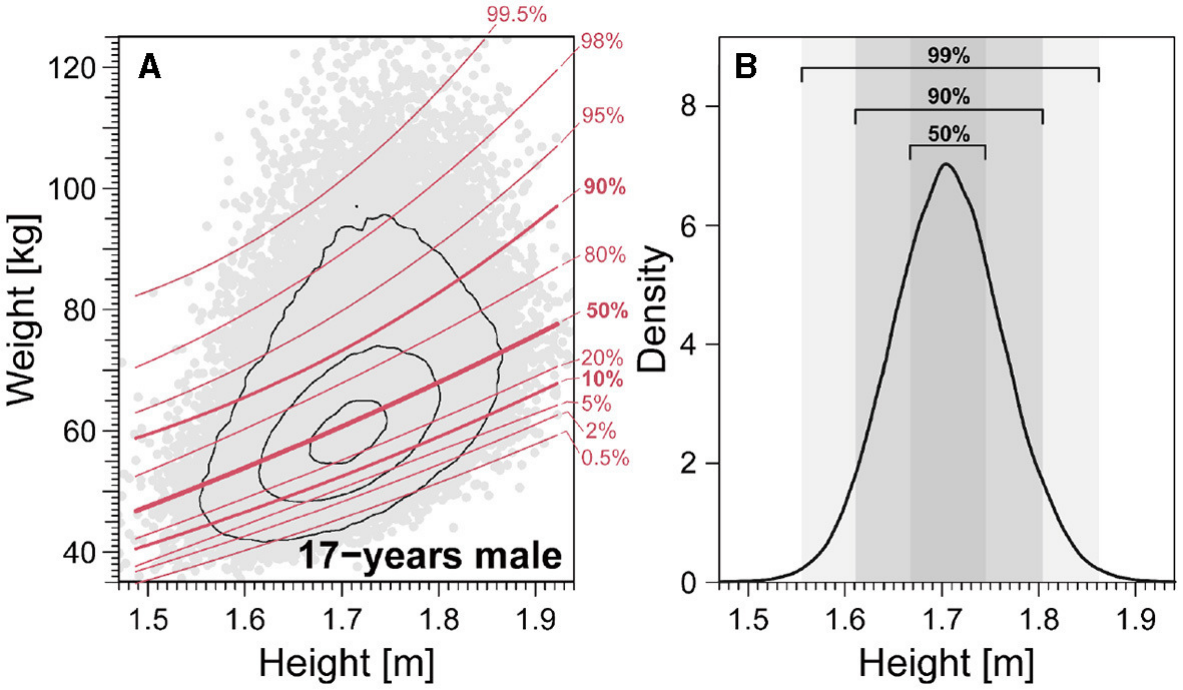
Quantile curves for weight-for-height distribution for 17-year-old males. **(A)** Weight-for-height distributions. *Red lines* denote weight-for-height curves for different centiles of the height distribution. **(B)** 50%, 90%, and 99% central regions of the height distribution. We analyzed data spanning 2008–2019.

**Figure 7:**
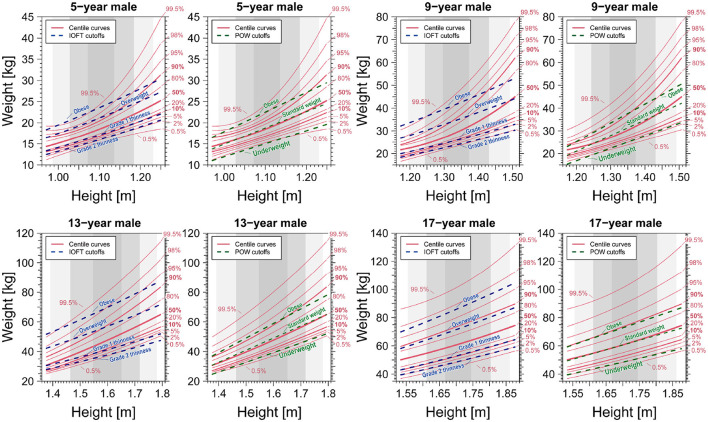
Comparison of centile curves of weight-for-height distribution and cutoff curves based on the BMI-based IOFT and POW criteria for males. We analyzed data spanning 2008–2019.

**Figure 8:**
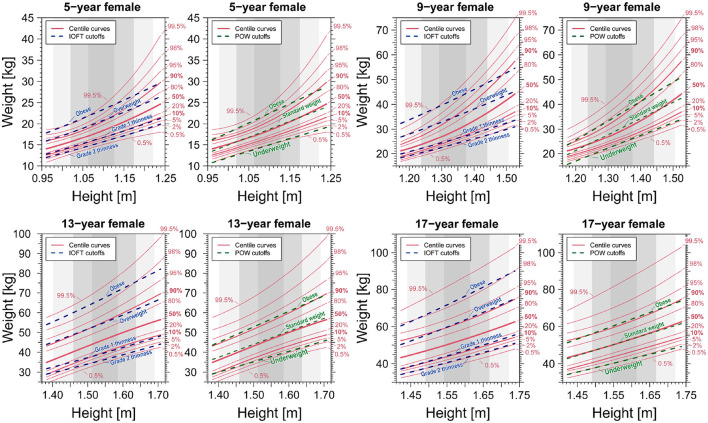
Comparison of centile curves of weight-for-height distribution and cutoff curves based on the BMI-based IOFT and POW criteria for females. We analyzed data spanning 2008–2019.

In the two bottom right panels of Figures 7, 8, we observe that centile and cutoff curves exhibit near-parallel alignment for males and females aged 17. However, for other age groups, a pronounced height dependence of the cutoff becomes evident. The obese cutoff intersects multiple percentile curves within the 80–99.5% range in the left panels showcasing IOFT cutoffs for 5-year-old and 9-year-old males in Figure 7 and females in Figure 8. This observation highlights a substantial height-dependent variation in the cutoff for these specific age groups. The POW criteria revealed shortcomings requiring attention, particularly in children under seven. One notable concern is the nonlinearity, represented by a curved shape, observed on the obese side of the centile curves (Figures 7, 8). This curvature introduces a height-dependent bias to the POW cutoff. Additionally, the cutoffs for underweight status fall below the 0.5% curve, indicating an excessively low threshold that warrants adjustment. Addressing these issues is crucial for refining the accuracy and reliability of the POW criteria, especially in assessing children under the age of seven.

### 3.2 Age dependence of 10th and 90th centiles in weight-for-height distribution

**Figure 9:**
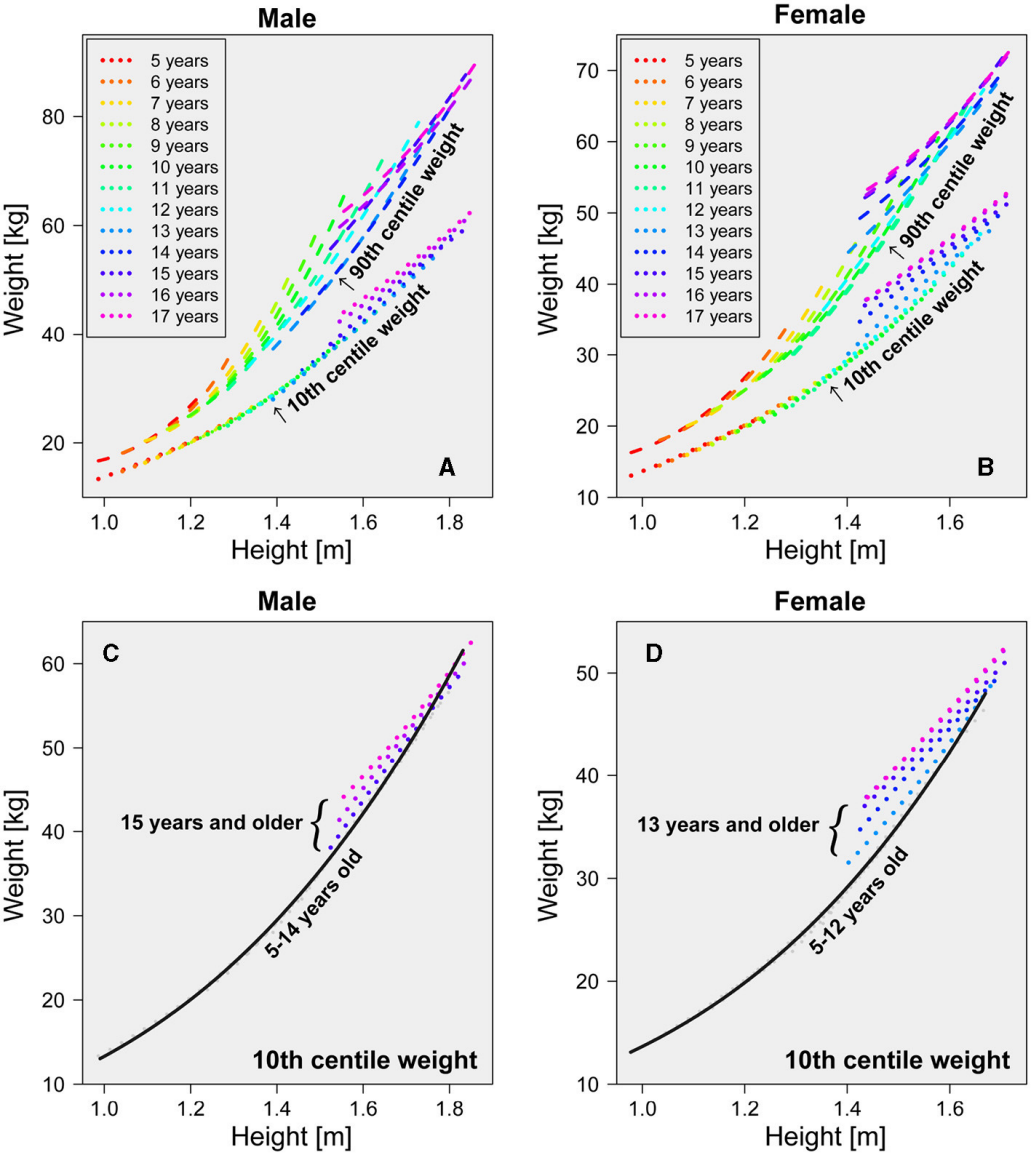
Age dependence of 10th and 90th centile weight. Each age's 10th and 90th centile weights are plotted by dotted and dashed lines, respectively. **(A)** 10th and 90th centile weight-for-height curves for males of all ages. **(B)** 10th and 90th centile weight-for-height curves for females of all ages. **(C)** 10th centile weight-for-height curve for males 15 years and older. **(D)** 10th centile weight-for-height curve for females 13 years and older. We analyzed data spanning 2008–2019.

When assessing thinness in young children, recommended cutoffs for weight-for-height distribution, irrespective of age, have been proposed. Notably, the National Center for Health Statistics/WHO weight-for-height reference (82) is among such references. We critically examined the applicability of weight-for-height-based assessments for thinness in children aged 5–17. In Figures 9A–D, the 10th and 90th centile boundaries depict the range encompassing 2.5–97.5% of height at each age, providing a comprehensive visualization of the thinness assessment across different age groups.

As illustrated in Figures 9A, B, the 90th centile curves linked to obesity reveal a clear dependence on age and height. Consequently, relying solely on age-independent weight-for-height relationships for assessing obesity becomes impractical. In contrast, the 10th centile curve associated with thinness exhibits an age-independent structure, particularly noticeable in males under 14 and females under 12, as illustrated in Figures 9C, D. Further insight from Figures 9C, D suggests that the growth process in children can be categorized into at least two distinct phases. The initial growth phase is characterized by a uniform cutoff boundary independent of age. Specifically, the 10th centile curves for males aged 5–13 and females aged 5–11 converge into a single curve. The solid lines represent an approximate curve derived from a cubic function for the 10th centile of the weight-for-height distribution in males aged 5–13 (Figure 9C) and females aged 5–11 (Figure 9D), providing a clear visualization of these distinct growth phases. The estimated function of the 10th centile cutoff for males was provided by


(3) $$\begin{array}{l} w=18.94h^{3}-24.00h^{2}+14.89h+3.74, \end{array}$$

and for females was provided by


(4) $$\begin{array}{l} w=15.82h^{3}-18.01h^{2}+12.93h+2.95, \end{array}$$

where *w* in Equations 3 and 4 represents body weight in kilograms (kg) and *h* represents height in meters (m). In the younger age groups, the cutoff obtained from the age-independent weight-for-height distribution (depicted by bottom *dotted lines* in Figures 9A, B) closely paralleled that established from the 10th centiles across each age bracket (depicted by bottom *solid black line* in Figures 9A, B). This alignment underscores the viability of using the lower centile of the weight-for-height distribution as an alternative method to delineate the cutoff within each cohort, specifically for males aged 5–13 and females aged 5–11.

The emergence of a second growth phase became noticeable in males aged 14 and older and females aged 12 and older. As depicted in Figures 9C, D, centile curves displayed a discernible upward shift within these age groups, indicating a dynamic progression. Consequently, the absence of a universally applicable cutoff curve independent of age became evident. The transition from the initial to the subsequent growth phases of the 10th centile curves might be attributed to the onset of secondary sexual characteristics in adolescence. Notably, as opposed to males, the 10th centile curves for females aged 16 and 17 exhibited striking similarity (lines overlap in Figures 9C, D), suggesting a near cessation of physical growth in females by age 16.

Suppose we consider using the rarity criterion based on the combination of weight and height to evaluate thinness and fatness; in that case, alternative assessment methods can be devised instead of relying solely on a BMI-based approach. For instance, the percentile position of a child could be quantitatively determined by plotting the measured weight and height on the centile curve space depicted in Figure 6 (refer to Supplementary Figures S1–S26; quick charts for the clinical assessment of thinness and obesity by sex and age). These graphical representations can assist clinicians and public health professionals determine a child's percentile position by graphing the measured weight and height on the centile curve space.

## 4 Discussion

Traditional assessments of thinness, underweight, overweight, and obesity have conventionally relied on the likelihood of weight/height combinations in the general population. In simpler terms, if a weight was observed with a perceived low likelihood of coexisting at a particular height, it prompted further scrutiny. This criterion lays the groundwork for the well-established BMI standard cutoffs for adults, a validity our analysis confirms for 17-year-olds approaching adulthood in both sexes. However, our quantile regression examination based on the IOFT criteria for underweight assessment unveiled marked height dependence in males aged 11–13 and females aged 10–11. Notably, a distinct bias surfaced at these specific ages, where children in the lower 25th percentile for height were classified as underweight five times more frequently than those in the upper 25th percentile. The overweight assessment also exhibited robust height dependence in males aged 8–11 and females aged 7–10. Children in the lower 25th percentile for height were deemed obese four or five times more frequently than their counterparts in the upper 25th percentile. Our findings highlight the complexities of age-dependent changes in body composition during the growth process in children, emphasizing the absence of gold standards for assessing underweight and overweight individuals in children and adolescents. Careful judgment is crucial in cases of short or tall stature at the same age.

A notable issue with the POW criterion is its ambiguous rationale: the POW distribution is age-dependent, making it impractical to use a constant value independent of age as the cutoff. The observed underestimation of the POW criteria, particularly in children seven and younger, is deemed unacceptable compared to the international BMI criteria assessment results. Therefore, at the very least, the POW cutoff should be modified to include age-dependent cutoffs to enhance its accuracy and relevance. The School Health Statistics Survey in Japan previously assumed that the weight-for-height distribution, categorized by sex and age, could be represented by a bivariate normal distribution, a regression line fitted to data within its 95% probability elliptic region modeled the standard weight-for-height relationship. However, our analysis—as depicted in Figures 7, 8—reveal that the weight-for-height distribution does not conform to a bivariate normal distribution for most children of both sexes. The standard weight-height relationship also exhibits a downward convex curve as age decreases. This challenges the mathematical expectation that all centile curves should be straight and parallel if a bivariate normal distribution approximates them (see Supplementary Figures S1–S26).

Underestimating the prevalence of underweight and obesity is particularly pronounced in individuals who are stunted or have shorter stature, and disparities in height due to geographical and ethnic variations further exacerbate this issue (83–89). The prevalence of stunting in children is alarmingly high, reaching 32% in Africa, 24% in Asia, and 11% in Latin America (90). Stunting is linked to severe adverse effects on child health and development, encompassing an elevated risk of morbidity, mortality, cognitive deficits, and potential long-term risk of metabolic syndrome in adulthood (91–95). The WHO has set an ambitious goal to reduce by 2025 the number of stunted children by 40% (96). However, this target may face formidable challenges, particularly in regions like sub-Saharan Africa, where the projected decrease in stunting prevalence is a mere 6% (from 38% to 32%) by 2025, with the actual number of stunted children expected to remain unchanged (97). On the opposite end of the spectrum, childhood obesity has reached epidemic proportions in developed countries, with 25% of US children being overweight and 11% classified as obese (98–100). Disturbingly, a notable 70% of obese adolescents are at risk of transitioning into obese adults (101, 102). The upward trajectory of childhood obesity has been consistent in developed nations since 1971 (98). Notably, obesity is linked to no less adverse outcomes in adulthood (103–105). This suggests that policies incorporating even slight discrepancies in the assessment of underweight and overweight status will affect a significant number of children and adolescents. To avoid this, it is crucial to accurately assess underweight and overweight status in children and adolescents with short stature. Assessing underweight and overweight status in childhood and adolescence is vital to surveillance and prevention efforts. The present study provides a framework that underscores the importance of considering age, height dependence, and variations when making such assessments.

Improving growth and nutritional status during childhood and adolescence can positively influence current and future generations' wellbeing. Precise estimation of the malnutrition burden at a population scale is vital for guiding interventions and assessing progress over time. Although we commonly use international standards like those set by the IOTF, incorporating metrics such as BMI and POW in the case of Japan—both of which rely on weight-for-height relationship, there is a notable risk of arriving at misguided conclusions about adolescent prevalence estimates if the underlying methodological limitations of the indicators and references are not thoroughly considered. Notably, the present findings highlight a paradox in utilizing the weight-for-height relationship for assessing thinness and fatness in children and adolescents. The extremities of the weight distribution, representing individuals at the highest nutritional or clinical intervention risk, face the greatest potential for misclassification, especially during periods of rapid growth. Our results underscore the need for heightened caution in assessing significantly lighter and heavier individuals during the rapid growth phases of children and adolescents. We advocate against using a universally applicable metric for all ages and heights in this population; instead, a more stratified approach is warranted, considering the individual's height relative to their age.

Finally, the BMI criteria outlined by the WHO or the Centers for Disease Control and Prevention (CDC) exhibit slight variations in value compared to the criteria used by the International Obesity Task Force (IOTF) in our study. These discrepancies lead to minor parallel shifts of the criterion curve in the weight-for-height distribution, as illustrated in Figures 7, 8. Consequently, any criterion derived from BMI is subject to similar biases as those discussed in this study.

In conclusion, we advocate for a discerning approach when employing anthropometric indicators, such as weight and height, to classify growth and nutritional status during childhood and adolescence. Stressing the importance of a broader perspective, we call for systematically collecting and considering information beyond anthropometry in policy and program decision-making. While recognizing limitations, we do not advocate for the outright dismissal of the IOTF or POW cutoffs. While the global nutrition community grapples with challenges, encompassing malnutrition in underdeveloped and developing nations and the obesity epidemic in developed nations, striving for a single international growth reference capturing universal childhood and adolescent growth patterns may not be prudent. We encourage nutrition researchers to prioritize exploring improved methods for assessing underweight and overweight status, particularly considering the age and height dependence of weight distributions in diverse populations.

## Data availability statement

The data analyzed in this study is subject to the following licenses/restrictions: access to this data is controlled by Japan's Ministry of Education, Culture, Sports, Science, and Technology (MEXT) in accordance with the policy of the Japanese school health survey. The data are available with permission from Japanese MEXT.

## Author contributions

YI: Formal analysis, Methodology, Writing—original draft. SN-O: Conceptualization, Writing—review & editing. MI: Formal analysis, Writing—review & editing. JK: Formal analysis, Writing—review & editing. NN: Conceptualization, Supervision, Writing—review & editing. MK: Conceptualization, Writing—review & editing. HO: Conceptualization, Funding acquisition, Project administration, Writing—review & editing. MM: Writing—original draft, Writing—review & editing. KK: Conceptualization, Formal analysis, Methodology, Project administration, Supervision, Visualization, Writing—original draft, Writing—review & editing.
