# Supplementary material for: Age- and height-dependent bias of underweight and overweight assessment standards for children and adolescents
Source: Front Public Health. 2024 Apr 24;12:1379897. doi: 10.3389/fpubh.2024.1379897 (PMC11076850; doi:10.3389/fpubh.2024.1379897)
Supplement: Supplementary file 1 [file Presentation_1.pdf]

Weight-for-Height Centile Charts by Sex and Age

Figs. S1–S26 show the estimated weight-for-height centile curves by sex and age. Each chart illustrates the 0.5–99.5% and 2.5–97.5% height regions, shaded in light and dark gray, respectively. By plotting the observed height/weight combinations, these charts enable a quick estimation of the weight centile within the same sex, age, and height group. The purpose of these charts is to showcase the practical utility of this approach rather than to establish definitive criteria.

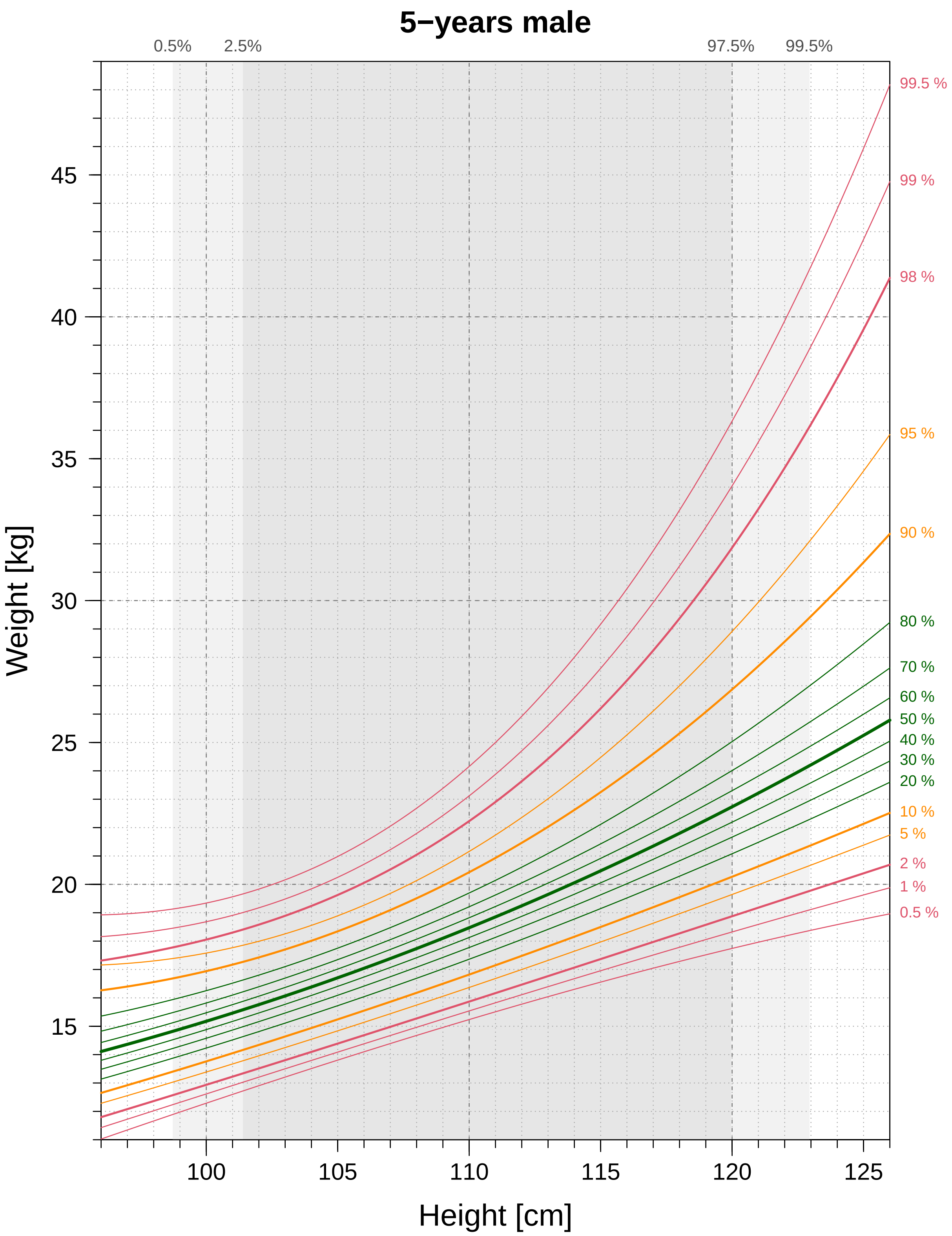

Figure S1. Weight-for-height centile chart for 5-year-old males.

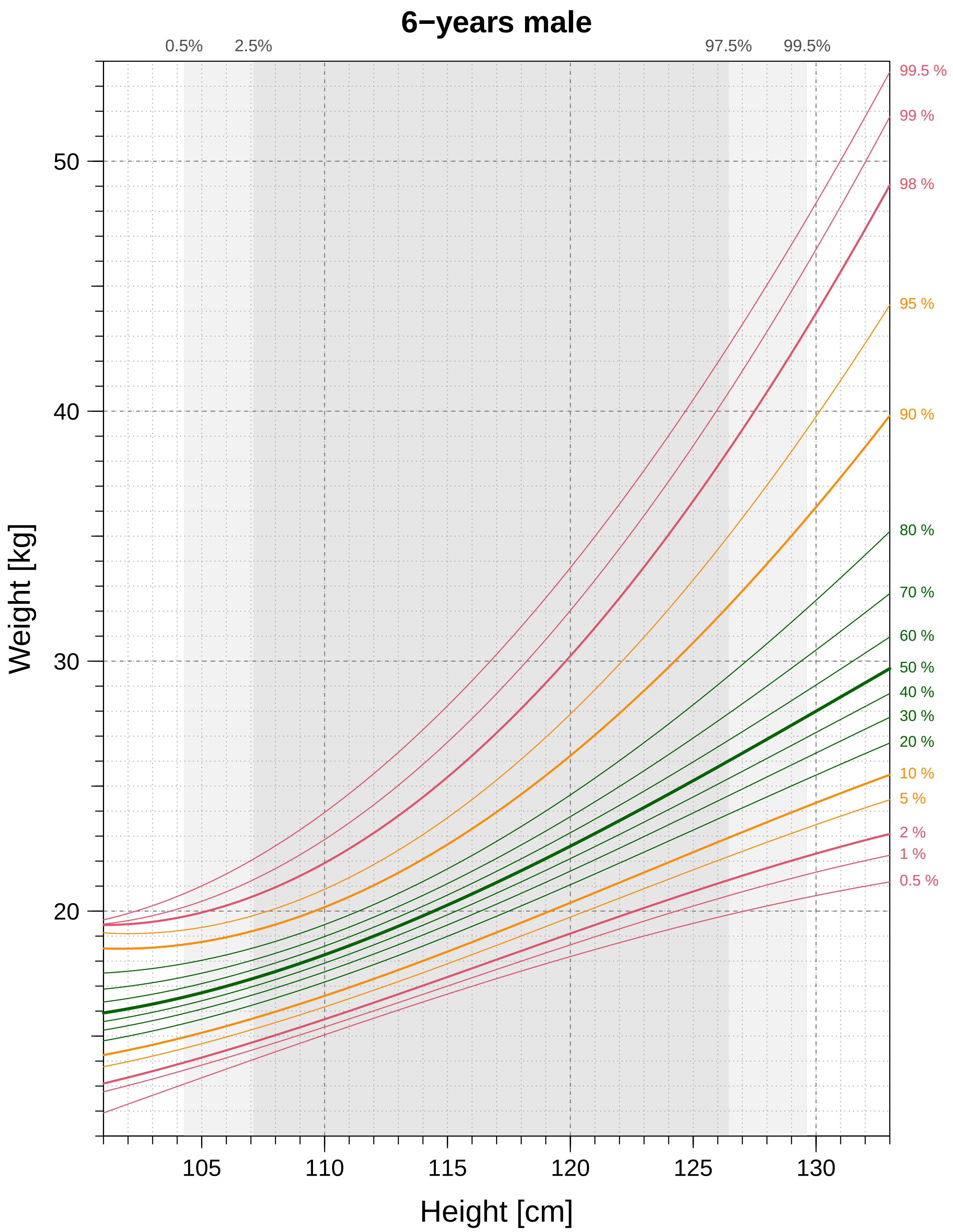

Figure S2. Weight-for-height centile chart for 6-year-old males.

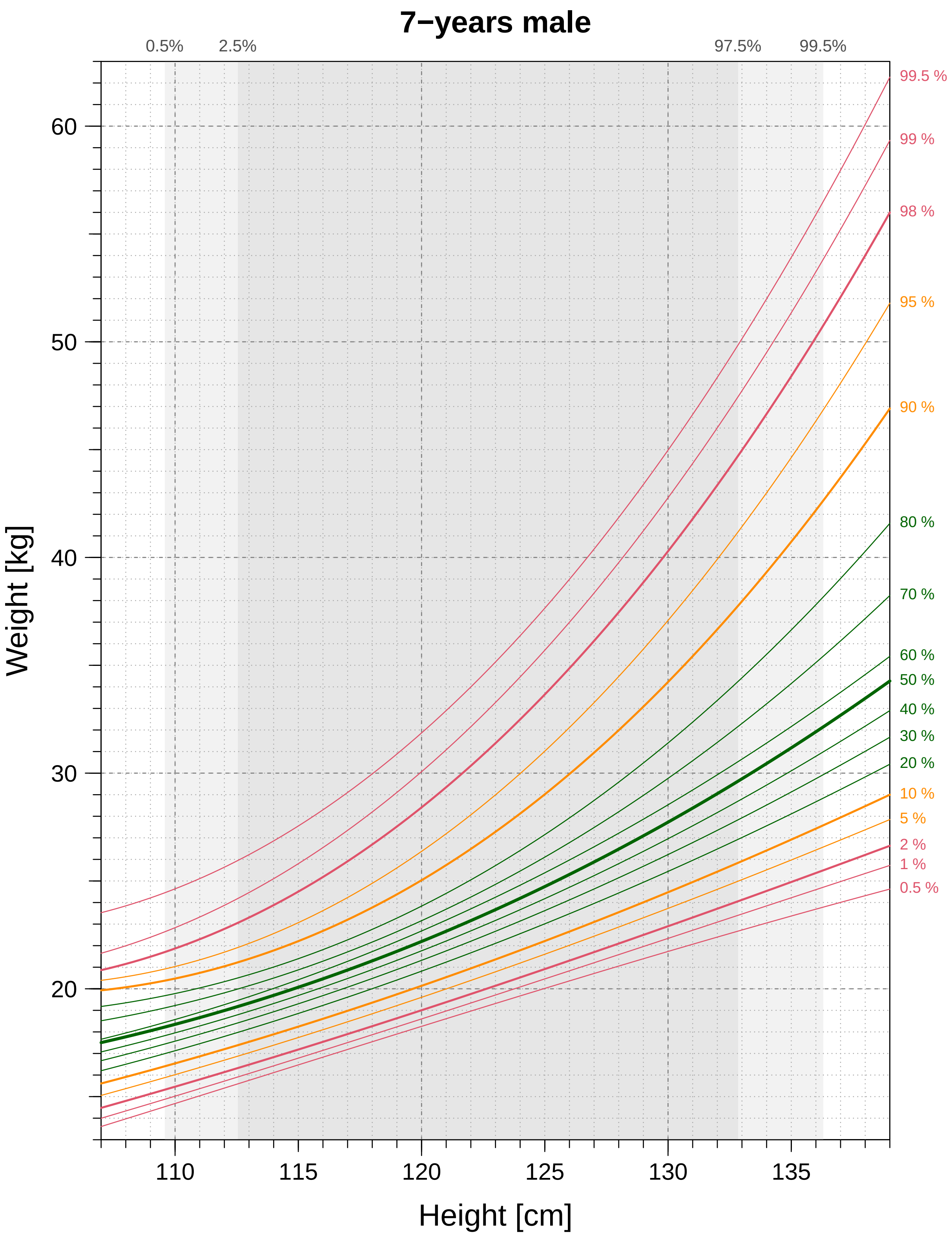

Figure S3. Weight-for-height centile chart for 7-year-old males.

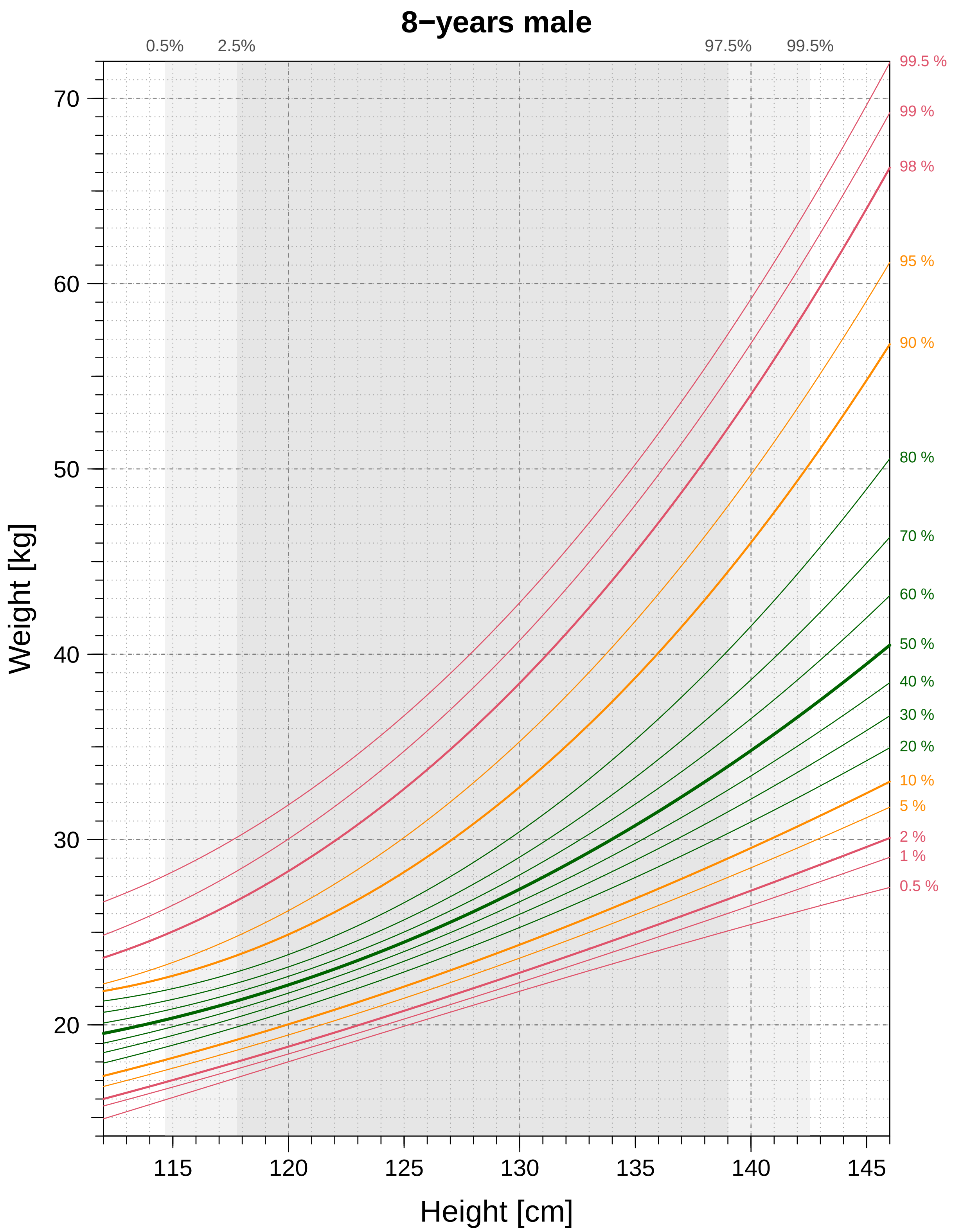

Figure S4. Weight-for-height centile chart for 8-year-old males.

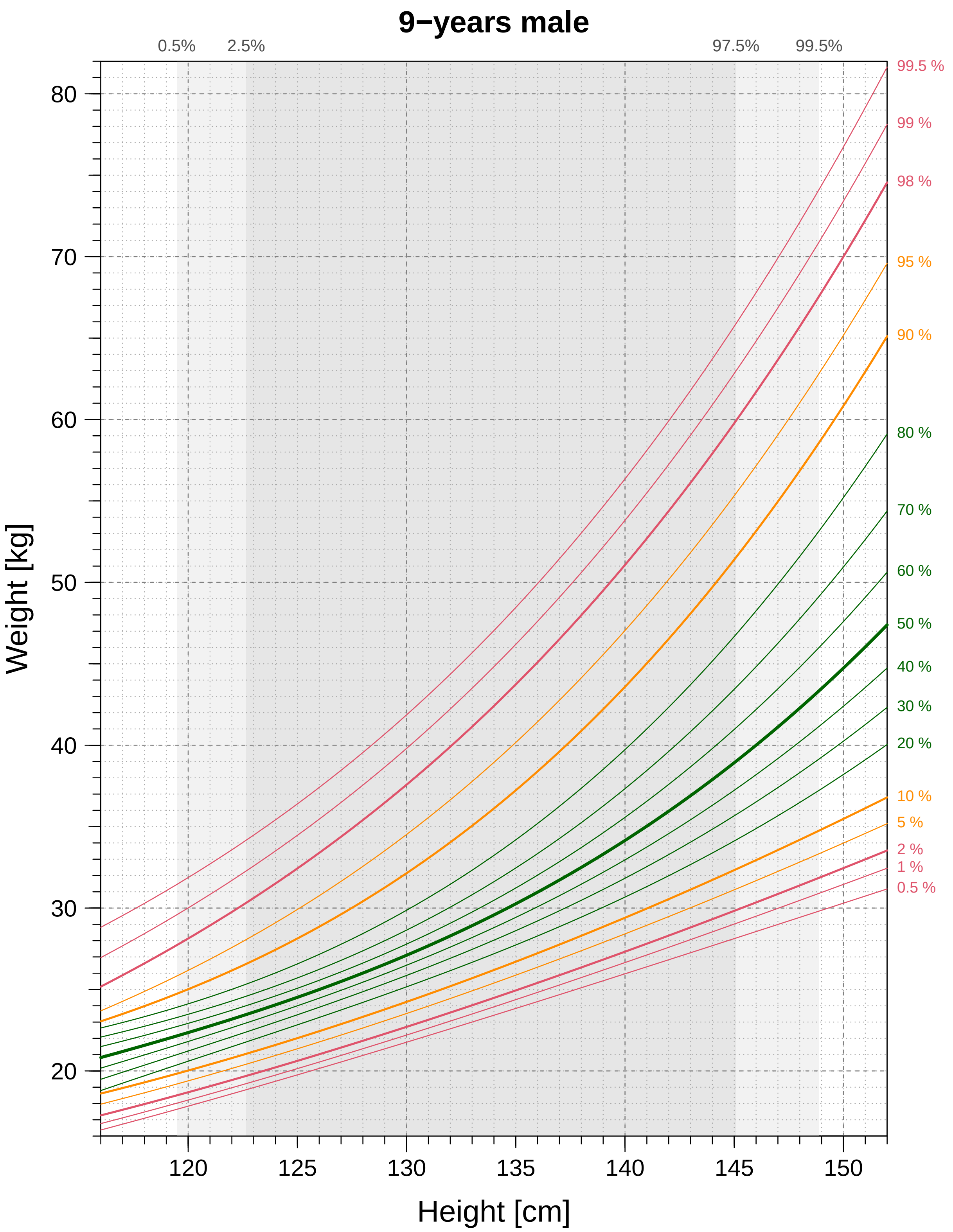

Figure S5. Weight-for-height centile chart for 9-year-old males.

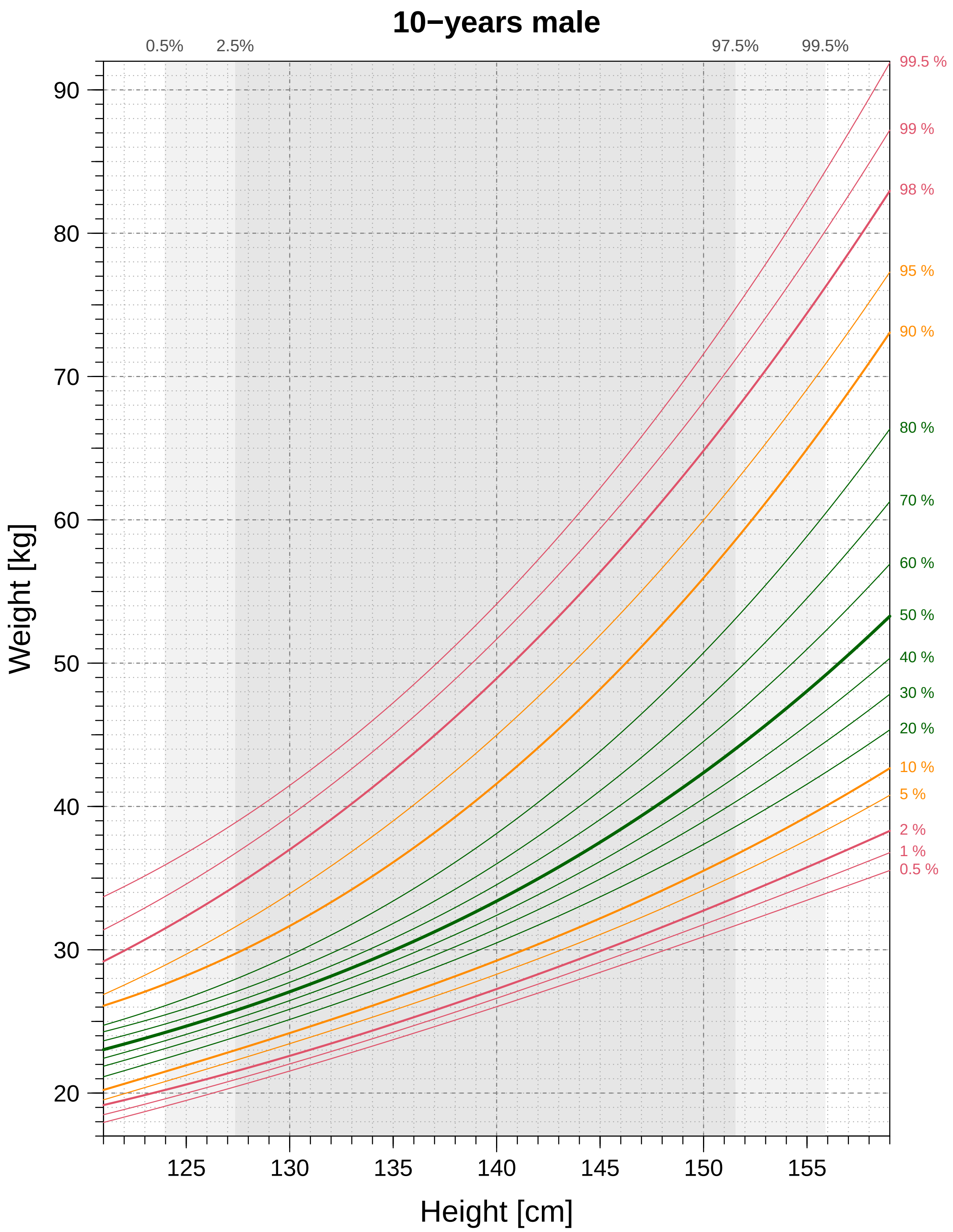

Figure S6. Weight-for-height centile chart for 10-year-old males.

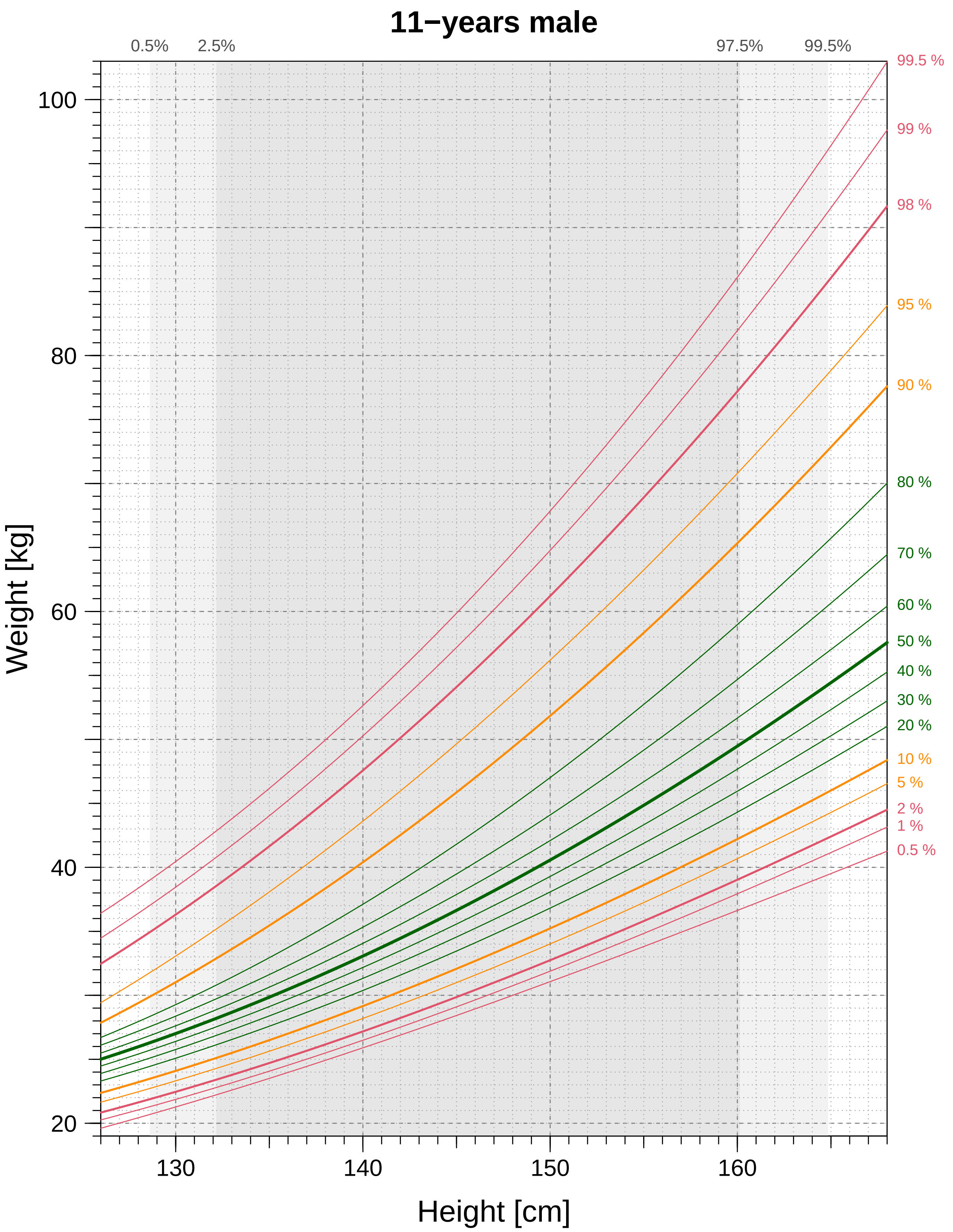

Figure S7. Weight-for-height centile chart for 11-year-old males.

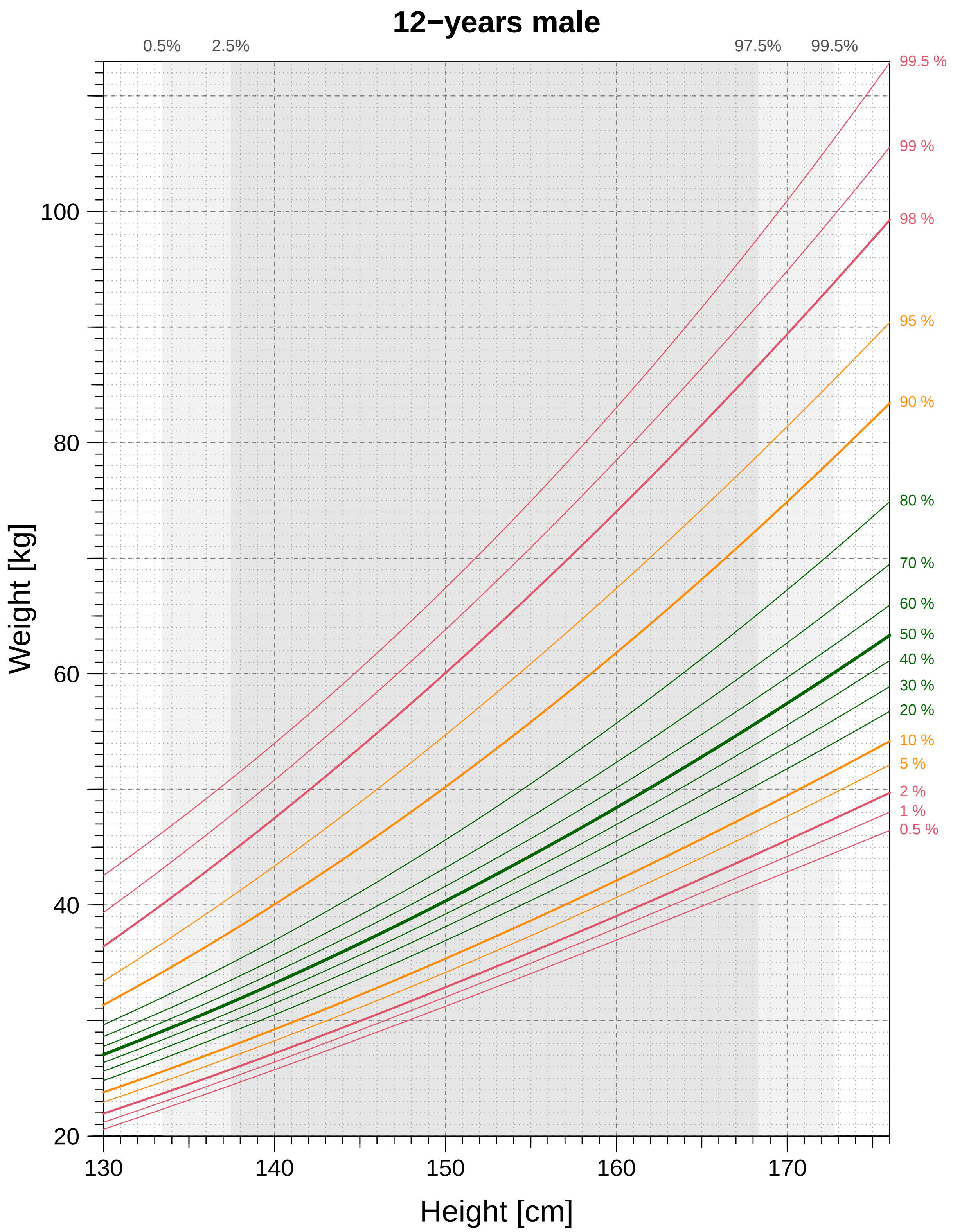

Figure S8. Weight-for-height centile chart for 12-year-old males.

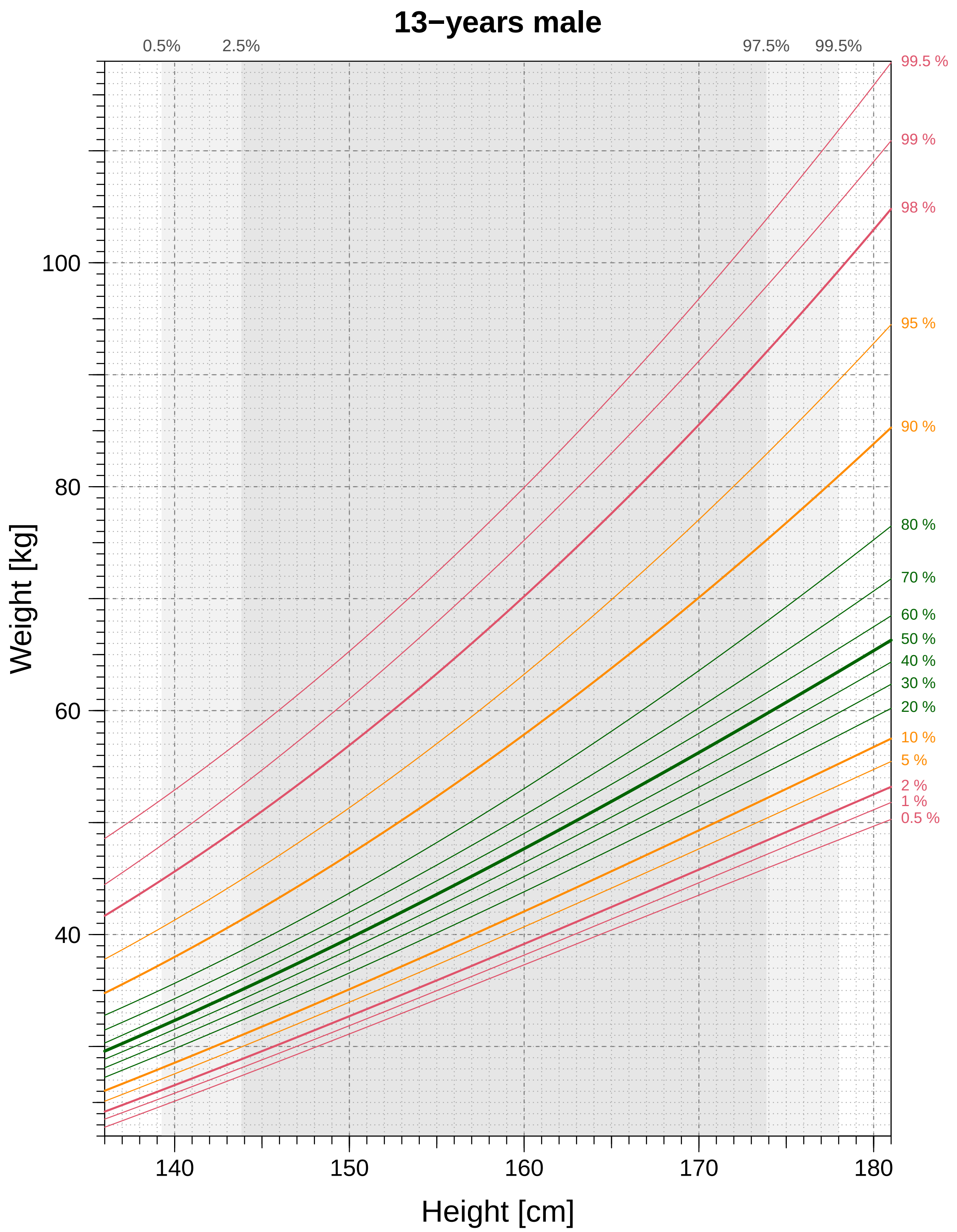

Figure S9. Weight-for-height centile chart for 13-year-old males.

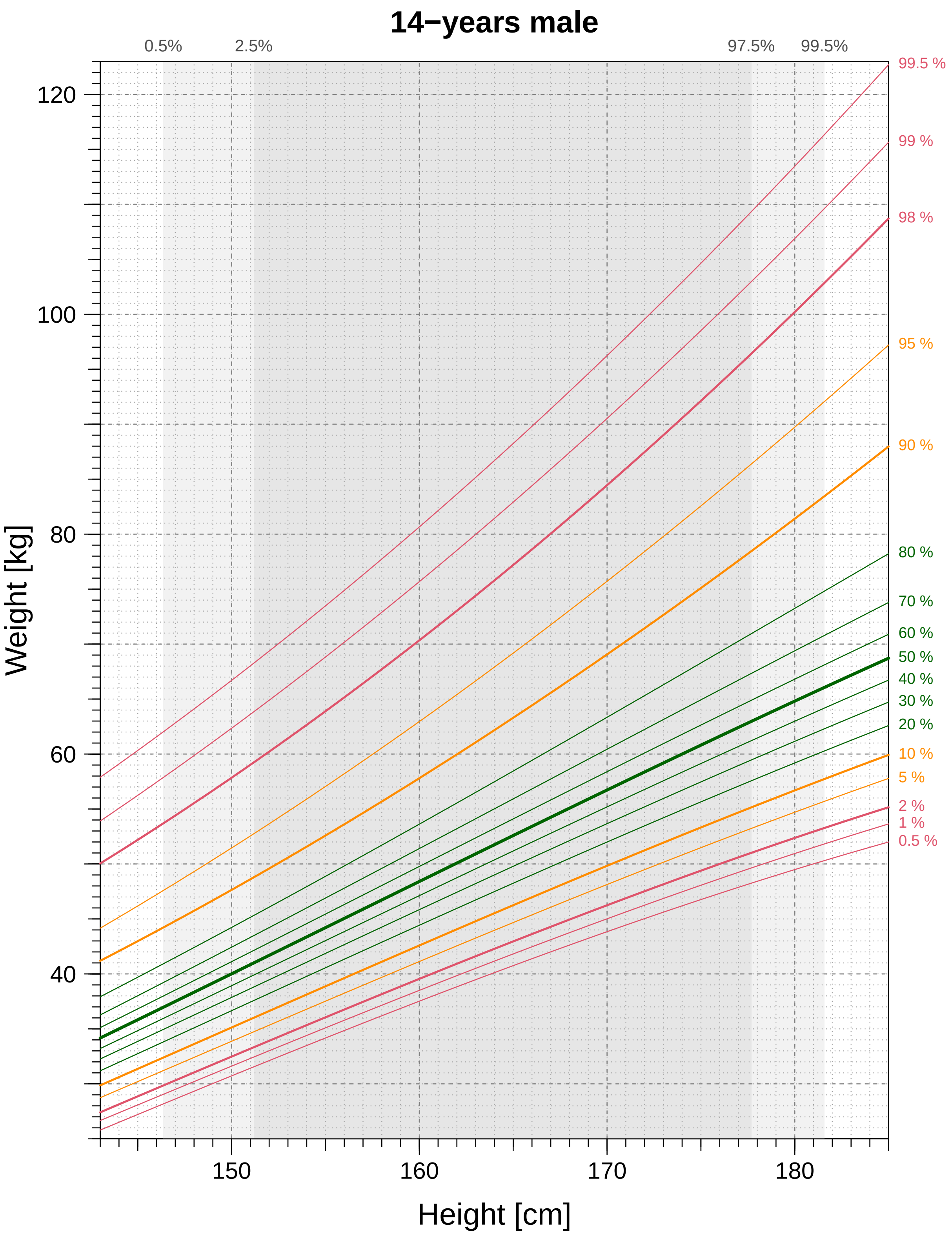

Figure S10. Weight-for-height centile chart for 14-year-old males.

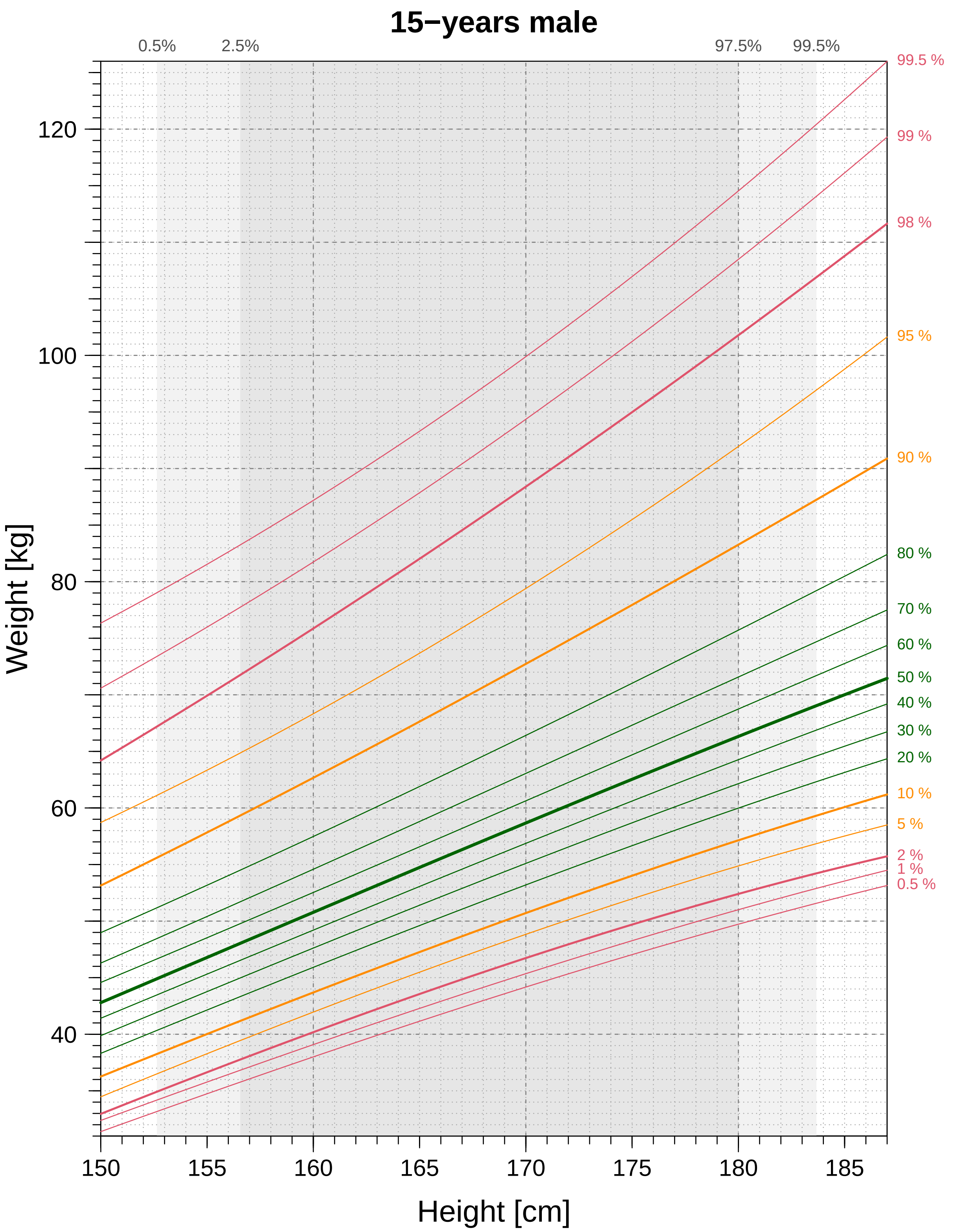

Figure S11. Weight-for-height centile chart for 15-year-old males.

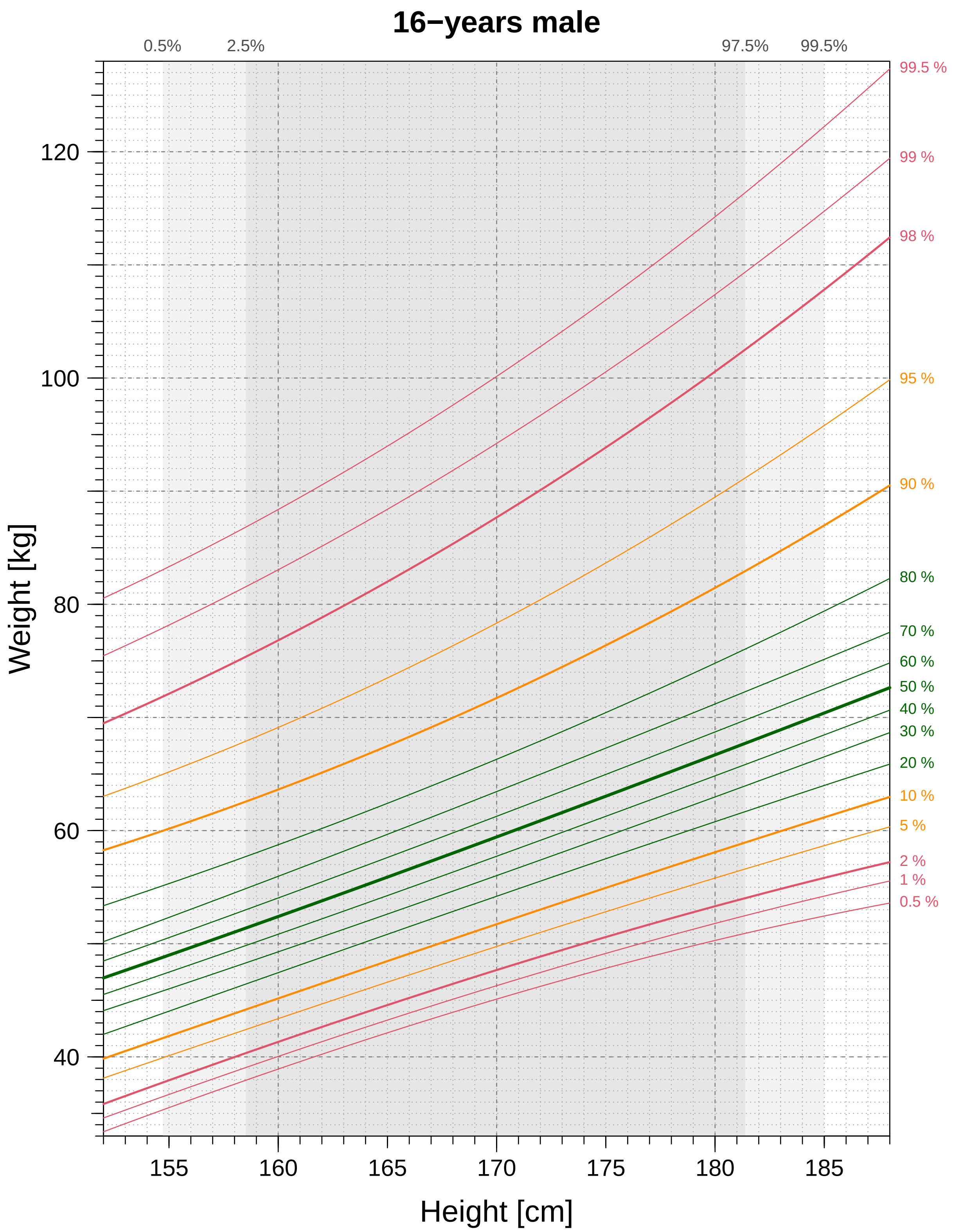

Figure S12. Weight-for-height centile chart for 16-year-old males.

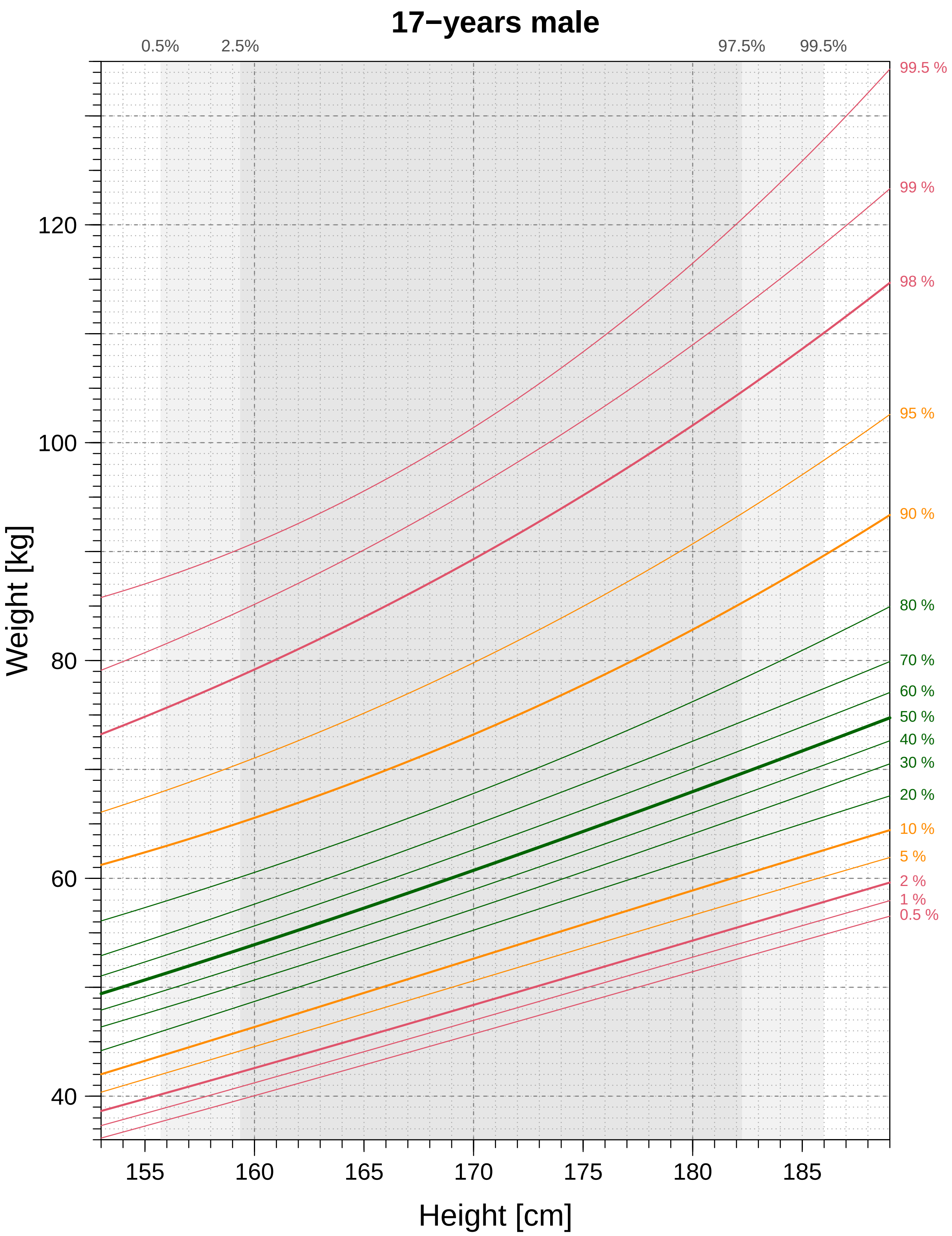

Figure S13. Weight-for-height centile chart for 17-year-old males.

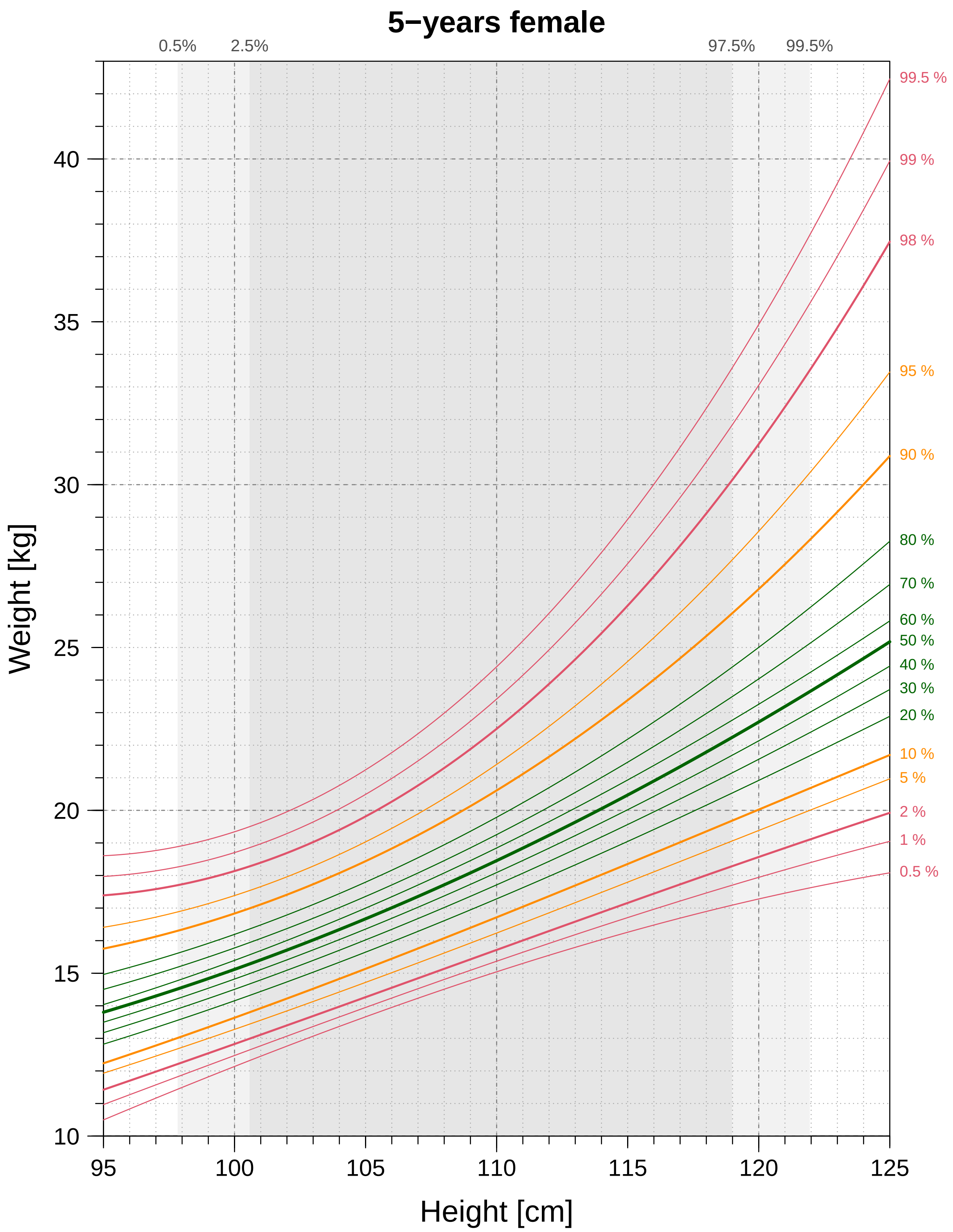

Figure S14. Weight-for-height centile chart for 5-year-old females.

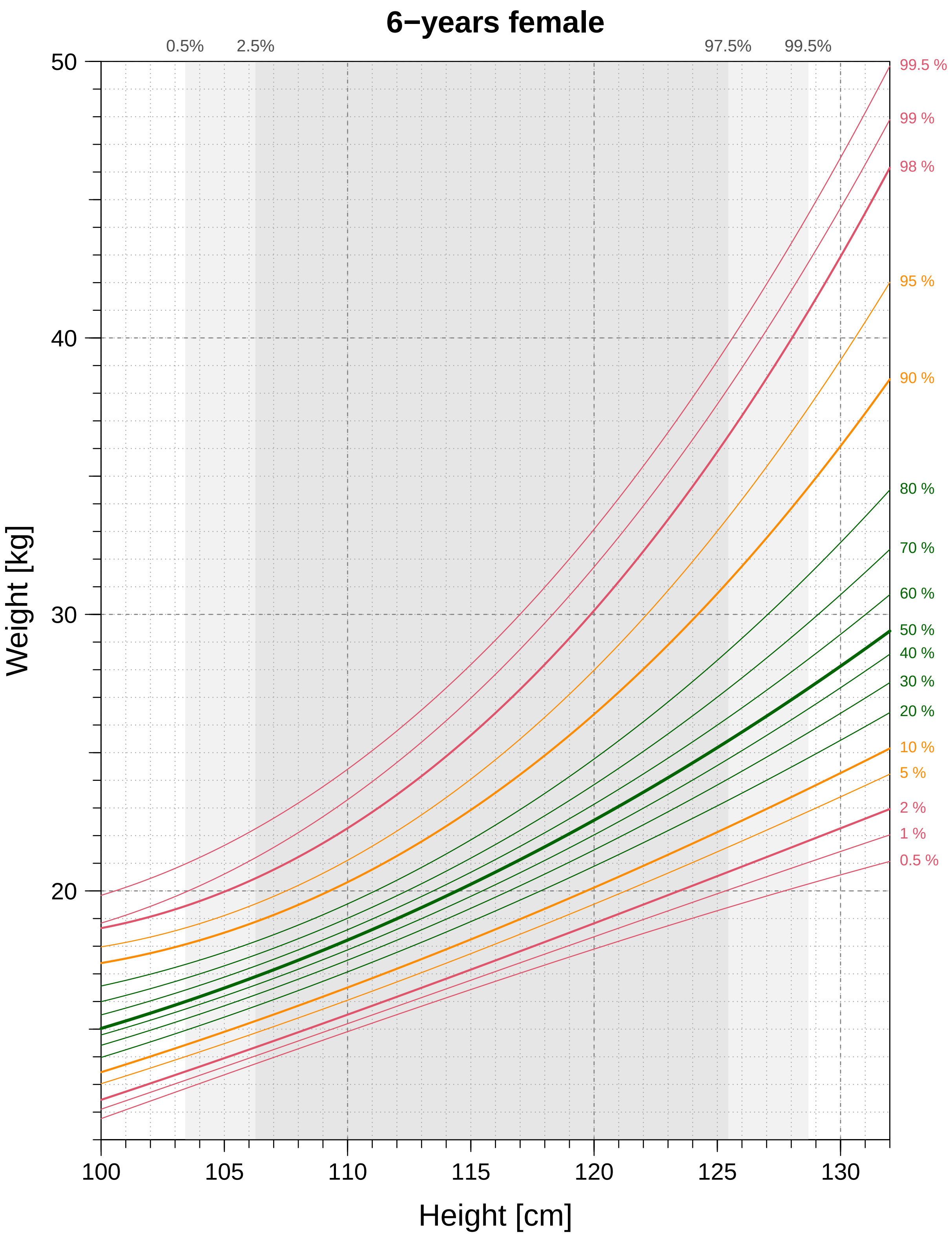

Figure S15. Weight-for-height centile chart for 6-year-old females.

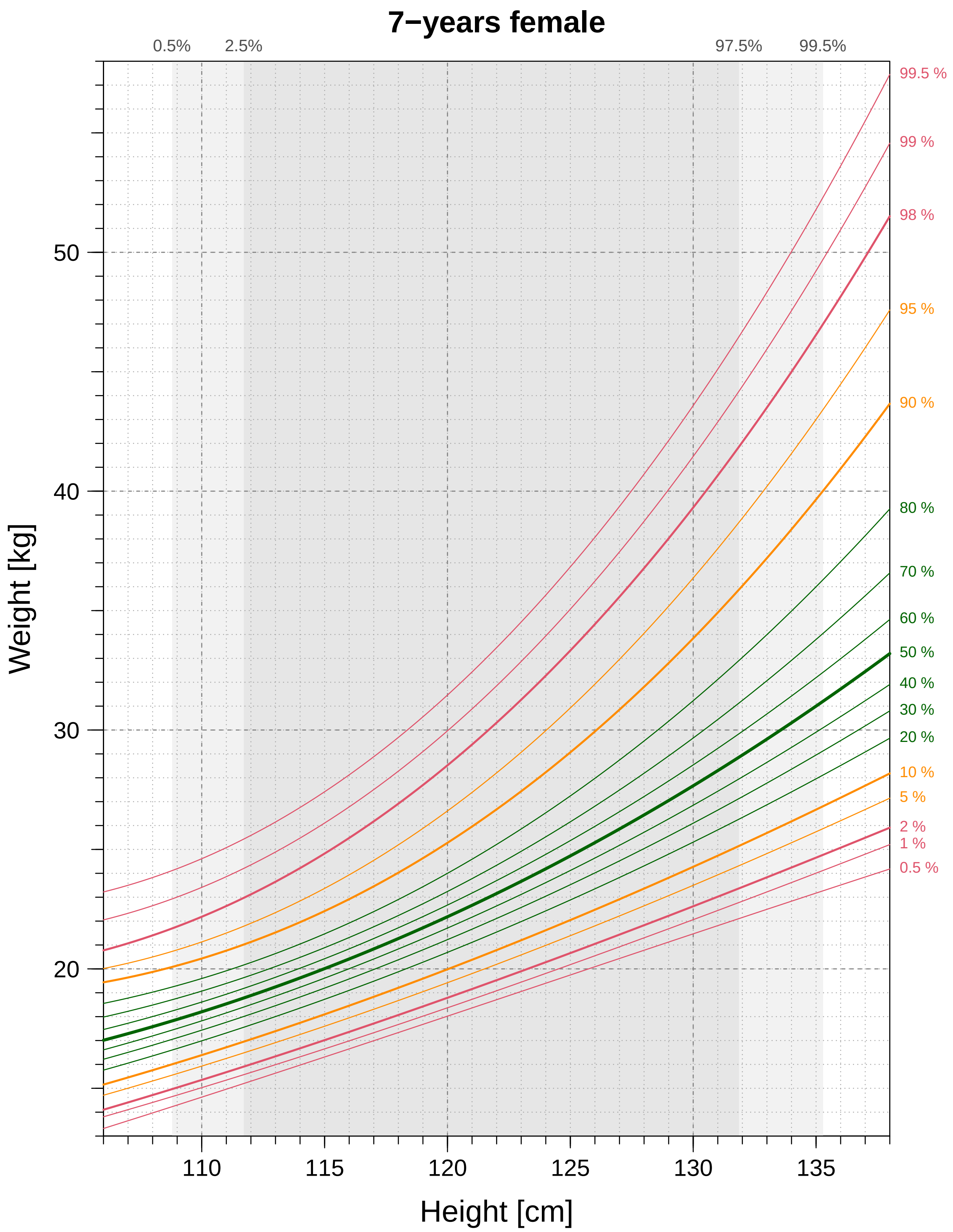

Figure S16. Weight-for-height centile chart for 7-year-old females.

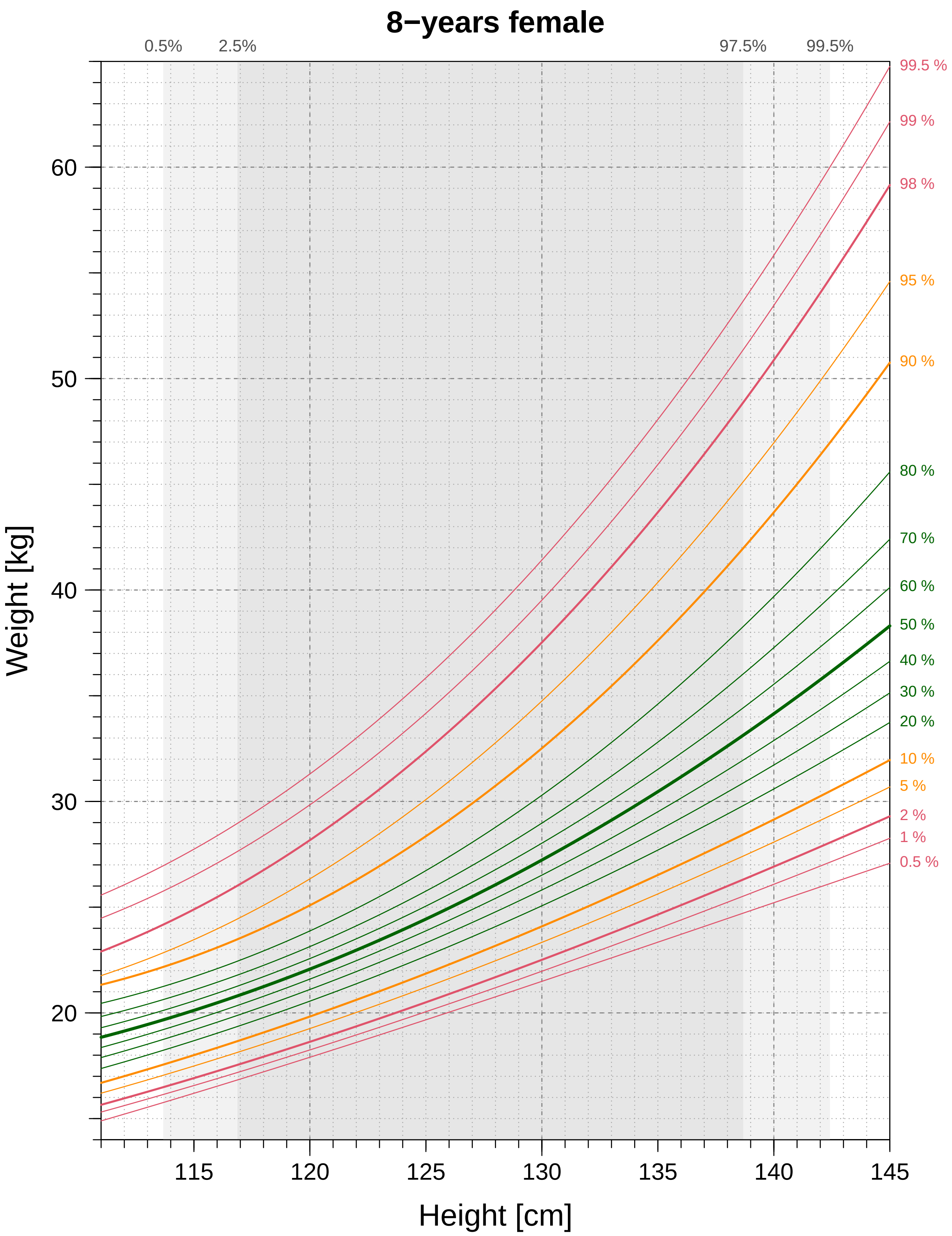

Figure S17. Weight-for-height centile chart for 8-year-old females.

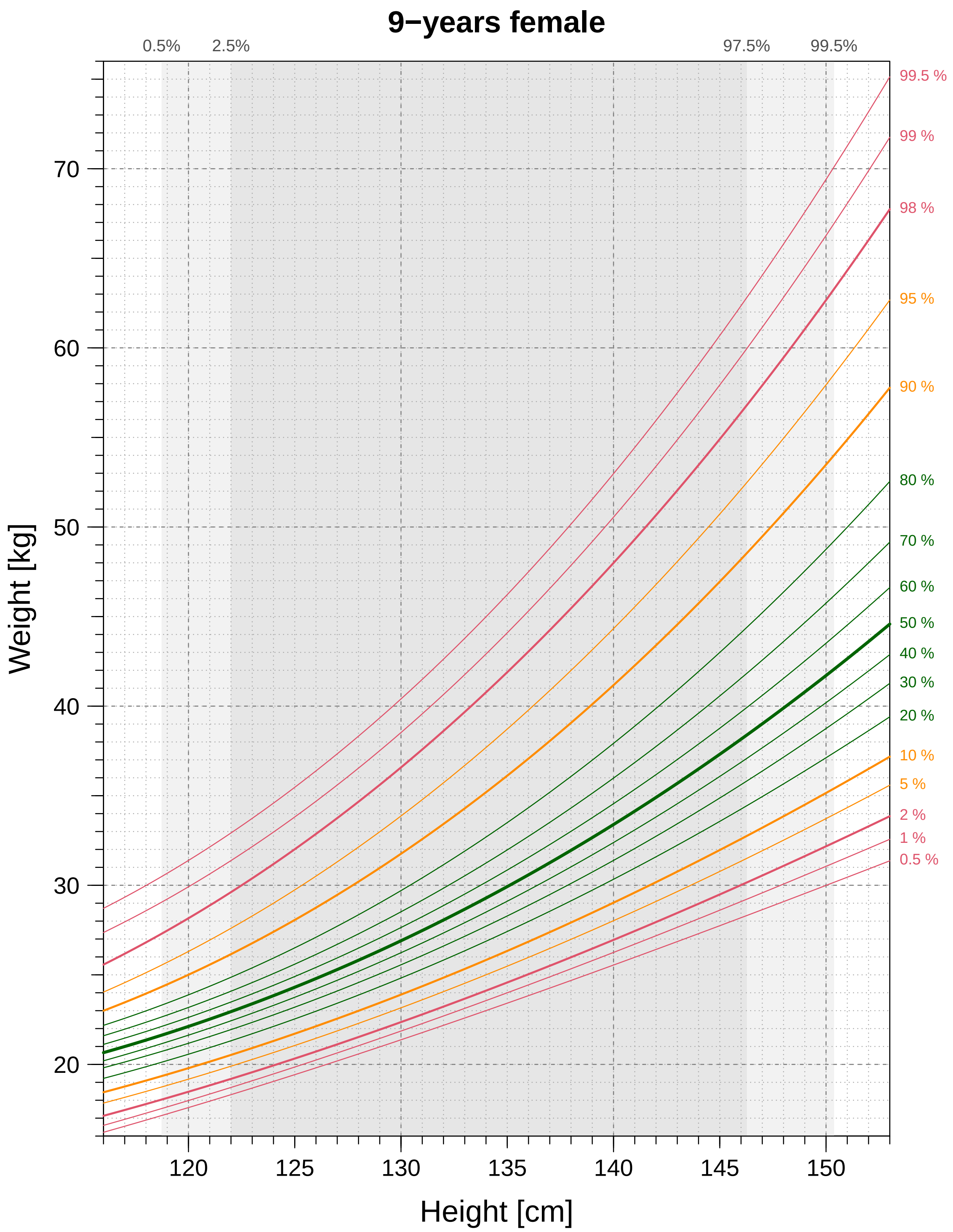

Figure S18. Weight-for-height centile chart for 9-year-old females.

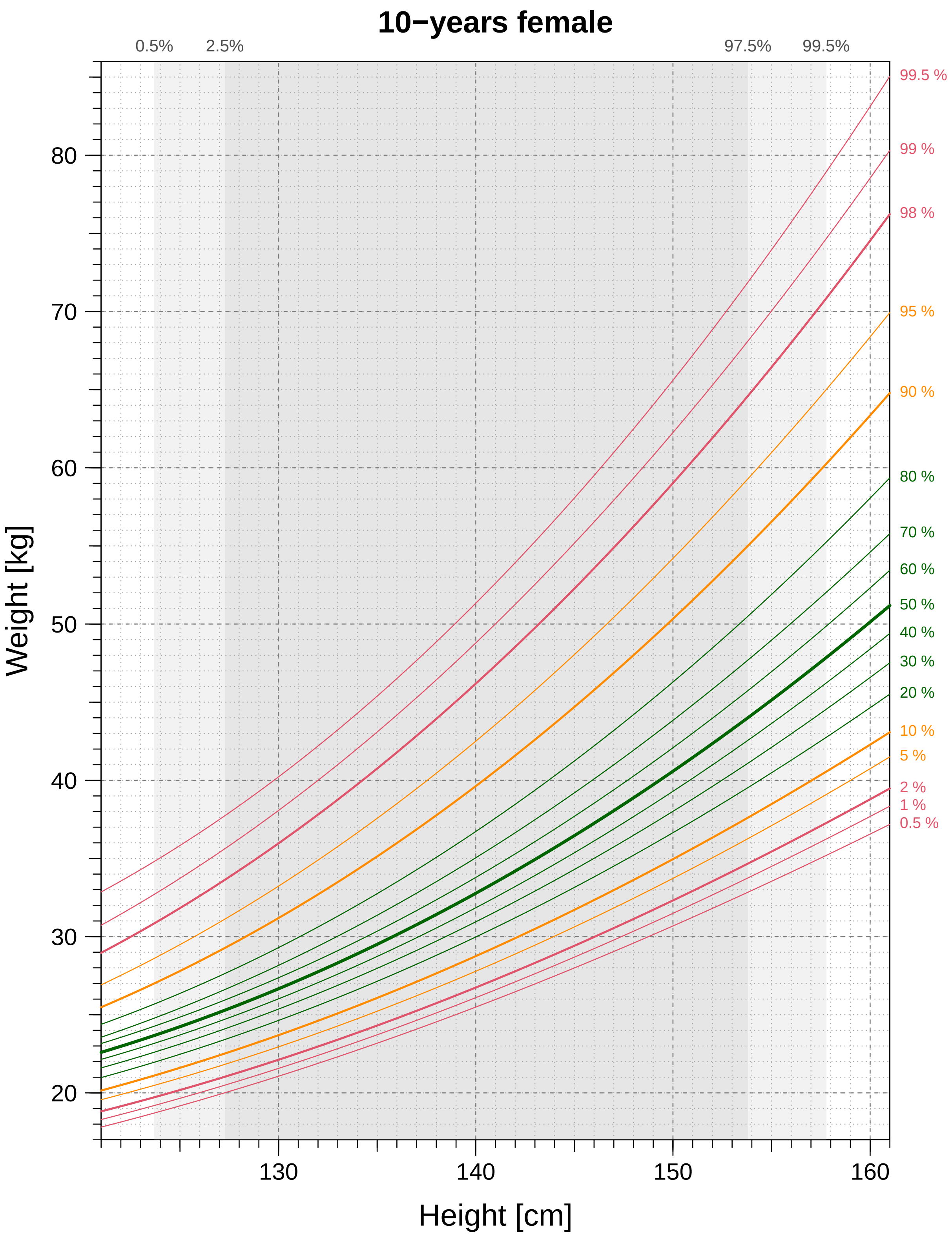

Figure S19. Weight-for-height centile chart for 10-year-old females.

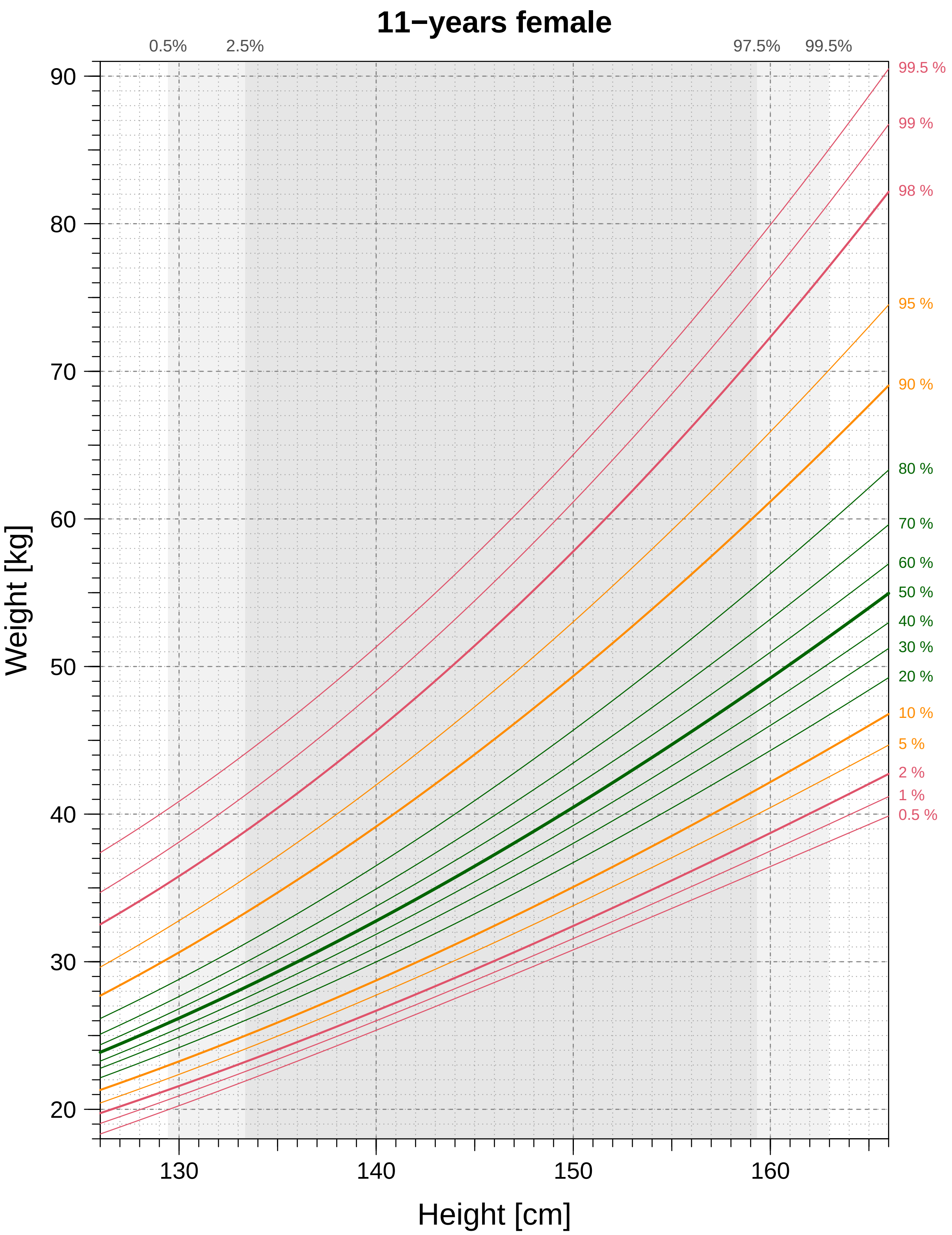

Figure S20. Weight-for-height centile chart for 11-year-old females.

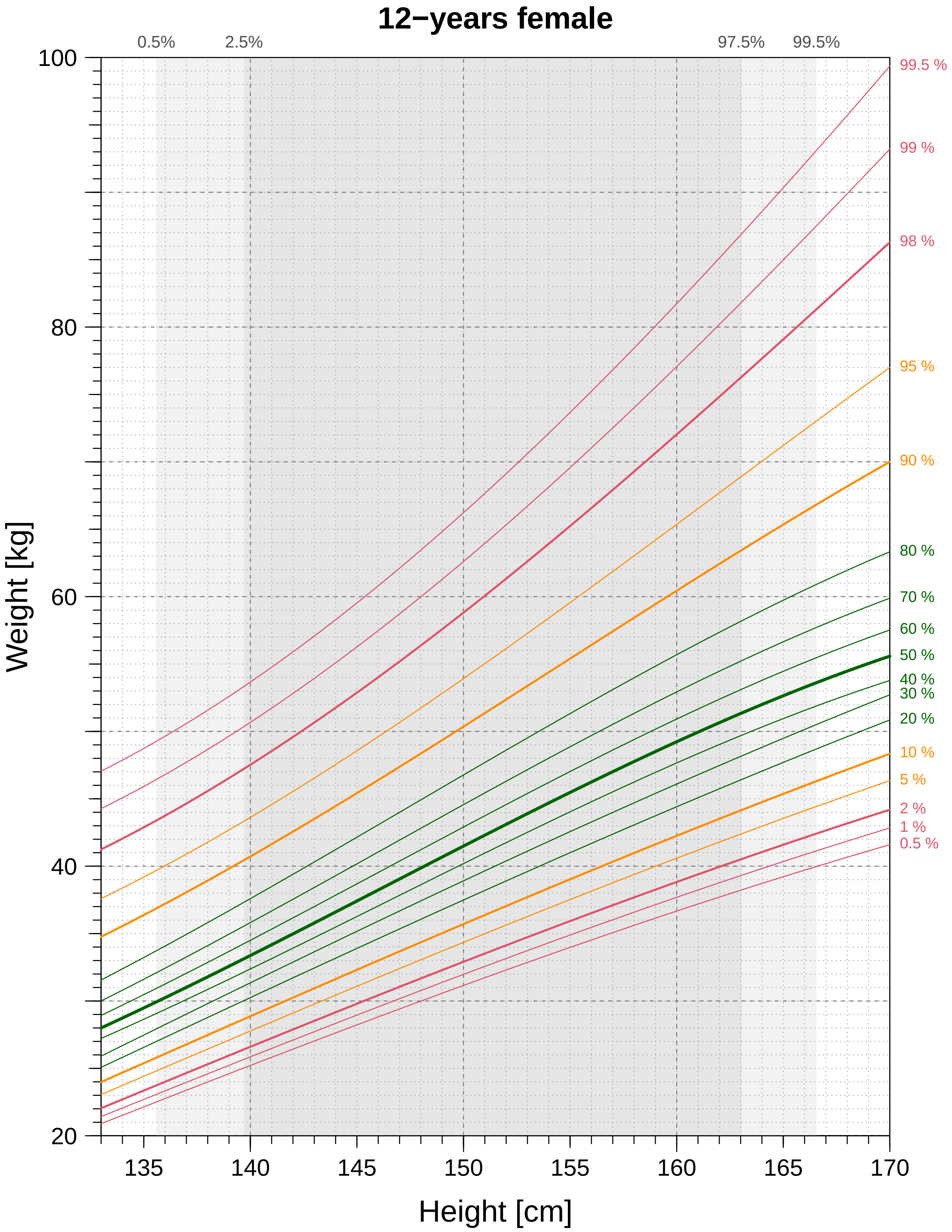

Figure S21. Weight-for-height centile chart for 12-year-old females.

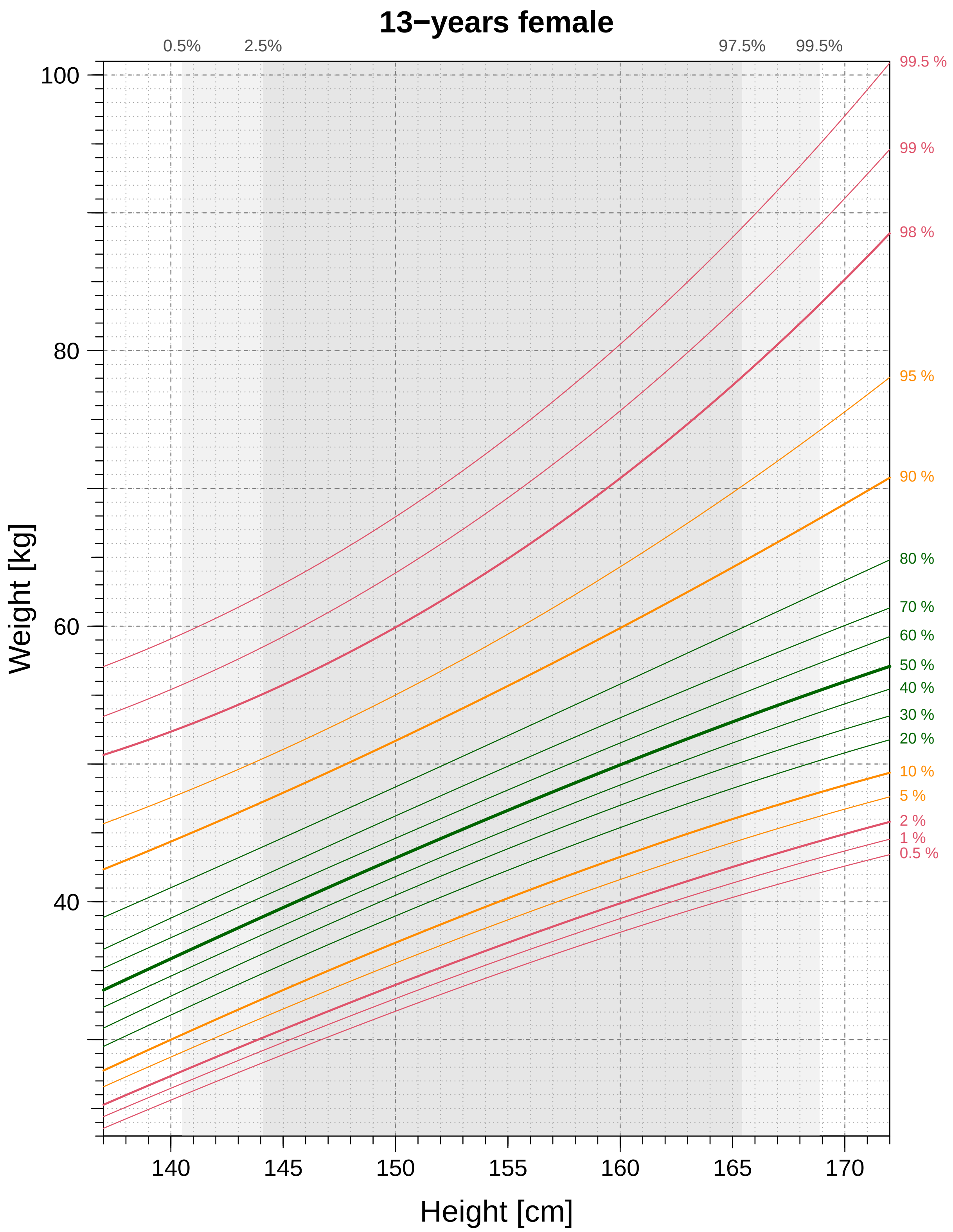

Figure S22. Weight-for-height centile chart for 13-year-old females.

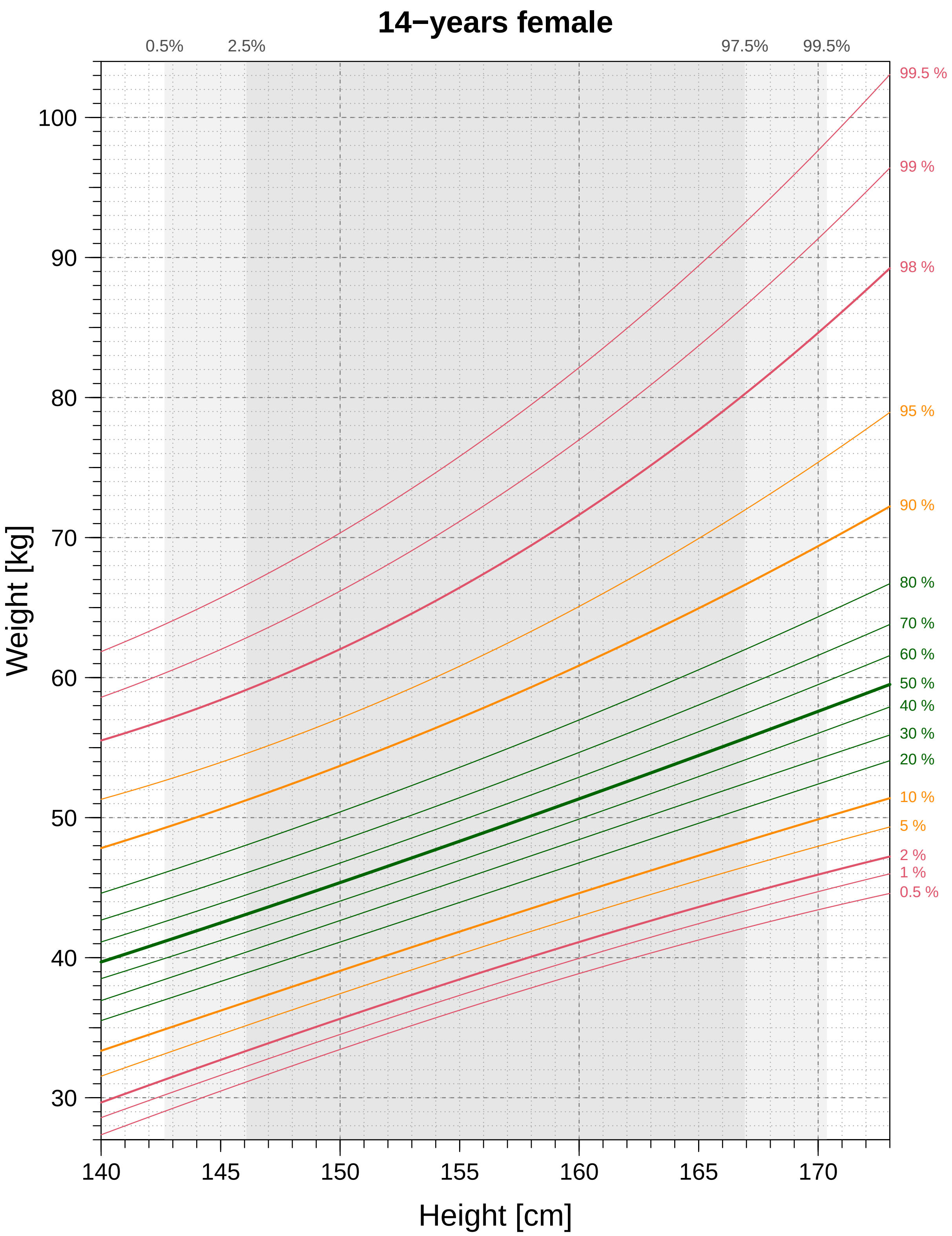

Figure S23. Weight-for-height centile chart for 14-year-old females.

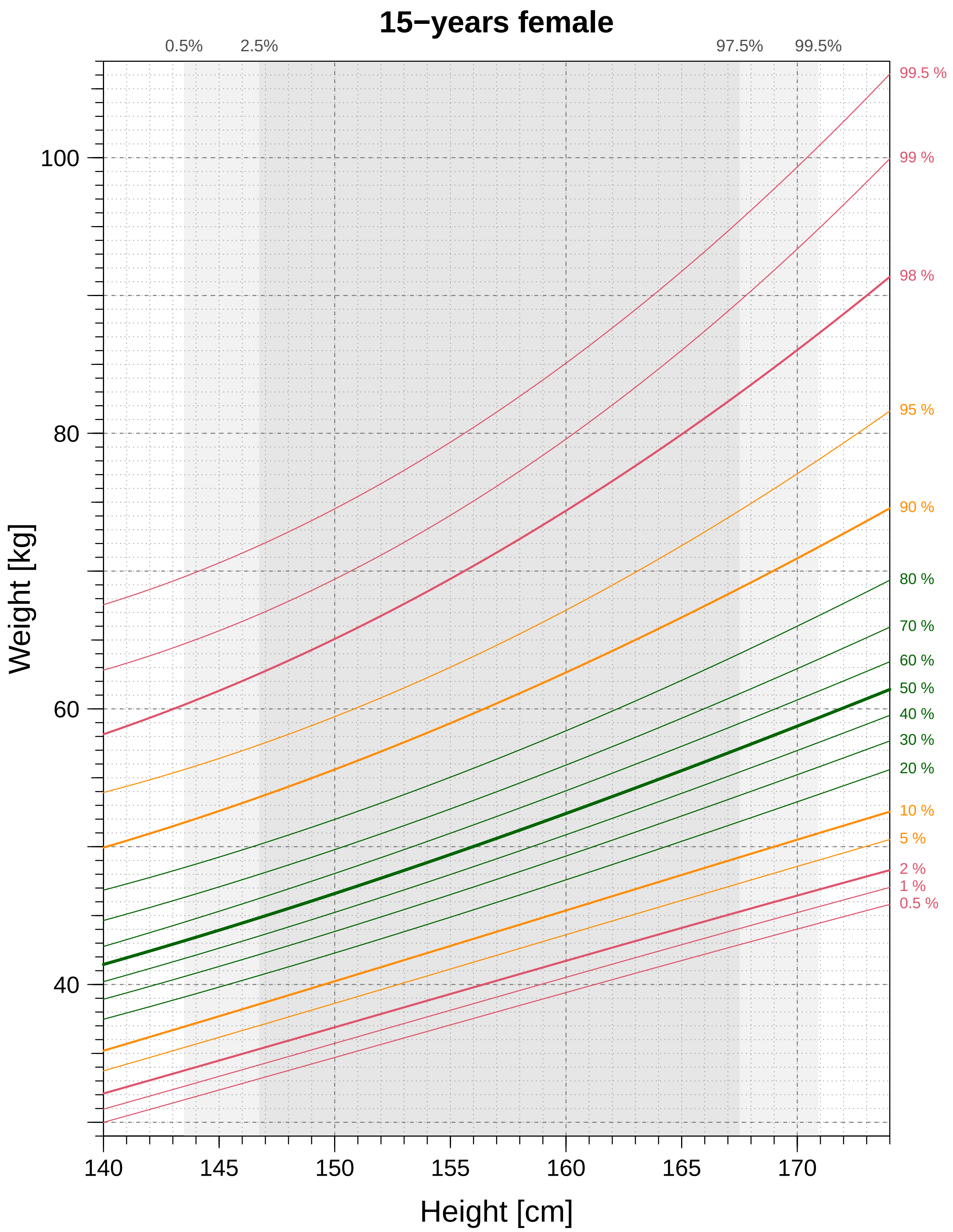

Figure S24. Weight-for-height centile chart for 15-year-old females.

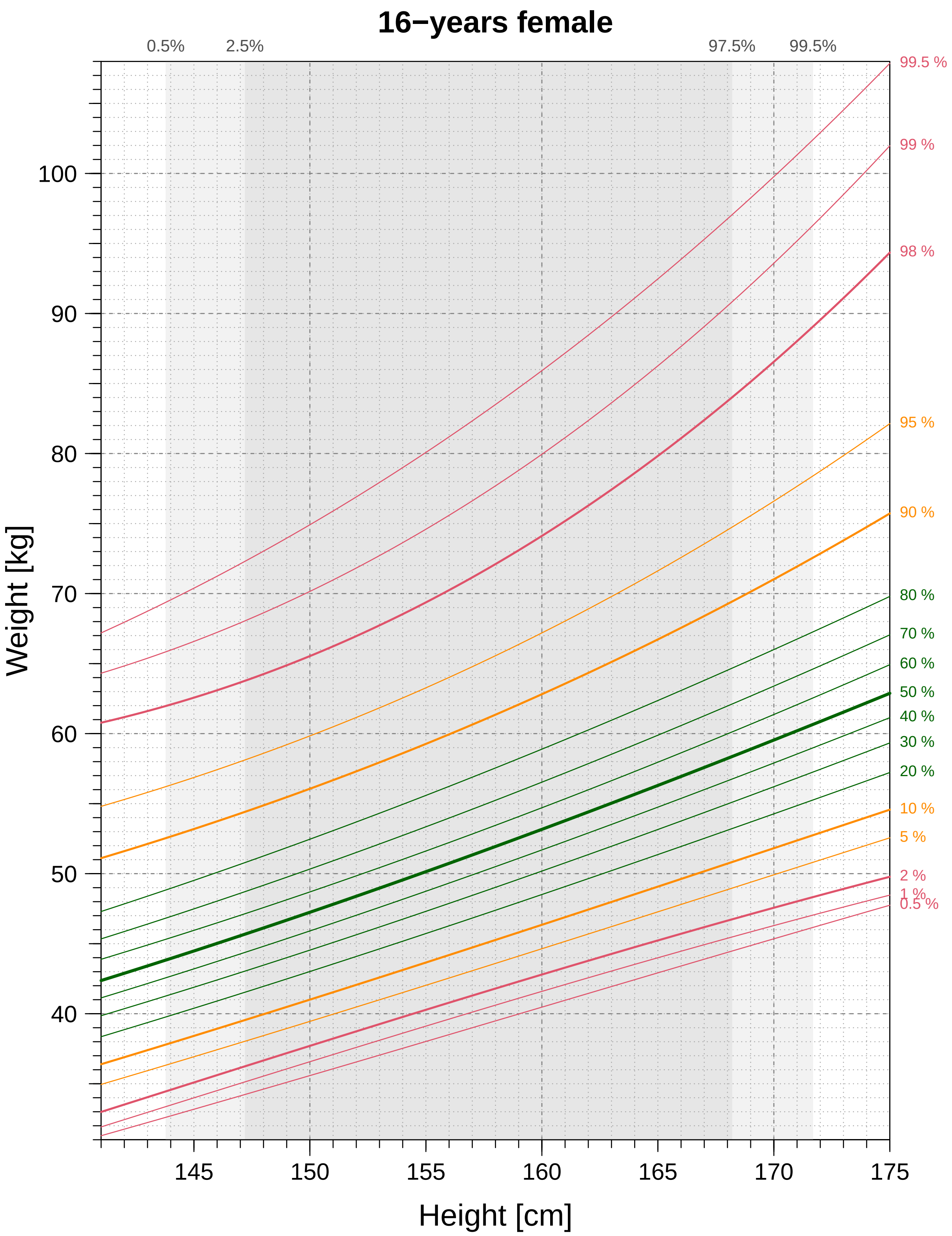

Figure S25. Weight-for-height centile chart for 16-year-old females.

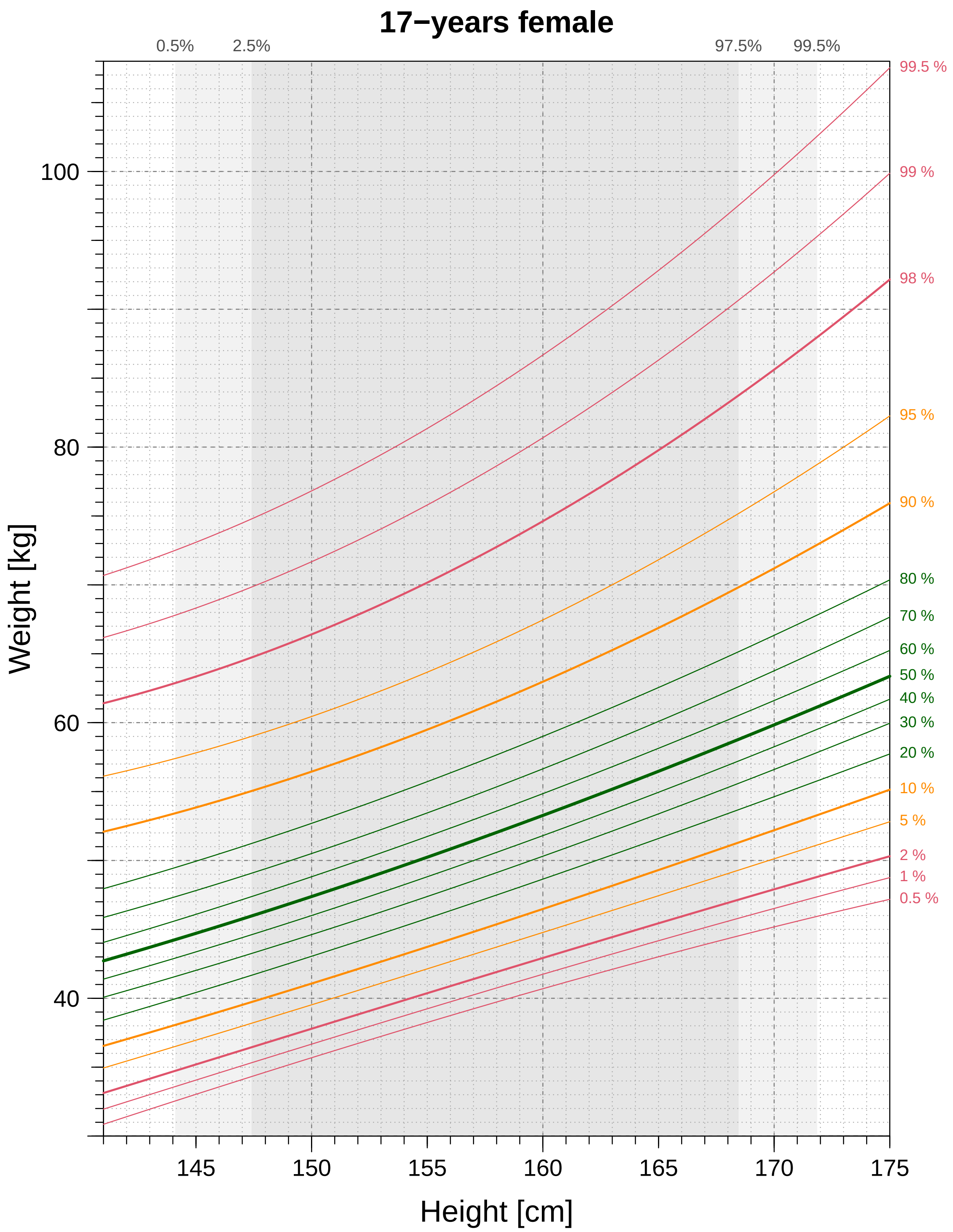

Figure S26. Weight-for-height centile chart for 17-year-old females.
